# Same ${{R}}_{0}$, Different Risk: Colonization-Age Timing and Heterogeneity Drive ICU MRSA Establishment and Burden

**DOI:** 10.1007/s11538-026-01770-9

**Published:** 2026-10-28

**Authors:** Taye Samuel Faniran

**Affiliations:** https://ror.org/043mz5j54 grid.266102.1 0000 0001 2297 6811 F.I. Proctor Foundation, University of California, San Francisco, CA USA

**Keywords:** MRSA, Intensive care unit, Colonization-age structure, Within-host-between-host coupling, Branching process, Reproduction number

## Abstract

Intensive care unit (ICU) control of methicillin-resistant Staphylococcus aureus (MRSA) occurs over short horizons, whereas $R_0$ summarizes average transmission at the importation-free disease-free state without encoding timing. We couple within-host bacterial and immune dynamics to an ICU model structured by colonization age, generating transmission and progression kernels that vary with colonization age. A two-stage framework uses a branching process to quantify finite-threshold crossing and a deterministic ward model to quantify conditional burden after the threshold is reached. Calibration fixes η=0.10 and distributes colonized admissions across pre-admission colonization ages. Of 1, 000, 000 parameter sets, 45, 250 satisfy cumulative $R^2\ge 0.90$. The representative calculation is subcritical, with $R_0=0.907957$; across accepted sets, the median and 95% behavioral range are 0.917464 and [0.814593,1.032975], with 92.460% below one. At $Z_{\textrm{crit}}=5$, exact threshold-reaching probabilities are 0.114659 for age-dependent transmission and 0.117074 for matched constant transmission. Their accepted-set difference range crosses zero, so this contrast has no robust direction. By contrast, age-dependent transmission reaches 50% of reproductive potential earlier in 98.378% of accepted sets. In 200-day conditional simulations, delaying $\tau _{\textrm{crit}}$ from 5 to 15 days increases the age-dependent peak total MRSA burden from 5.629 to 7.298 patients and cumulative total burden from 683.8 to 1398.0 patient-days. At the 9-day baseline and equal $R_0$, age dependence gives a 7.372% higher peak but a 9.304% lower cumulative burden than constant transmission. Increasing $\kappa _{\max }$ from 0.6 to 1.4 times baseline reduces $R_0$ by 4.133%. Thus, matching $R_0$ does not preserve timing-sensitive outcomes, but their magnitude and direction depend on the endpoint; over the examined ranges, clearance timing has greater leverage than immune-strength intensification.

## Introduction

Methicillin-resistant Staphylococcus aureus (MRSA) remains a persistent global cause of healthcare-associated morbidity, mortality, and cost. Its control is increasingly challenged by high patient turnover, heterogeneous colonization pressure, and constrained isolation capacity (Tobin et al. 2025; Elmi et al. 2026; Nelson et al. 2015; Bankar et al. 2024; Pal et al. 2024). Many standard infectious-disease models represent transmission using a constant per-capita rate during an infectious or colonized state. This time-averaged representation is useful, but it does not retain the distribution of transmission opportunities over time. Previous work has shown that the assumed timing and duration of infectiousness can alter epidemic growth and the interpretation of reproduction numbers (Wallinga and Lipsitch 2007; Wearing et al. 2005). In an ICU, this timing can also affect short-run dynamics because discharge, decolonization, treatment, and recovery may remove patients before later transmission opportunities are realized. Therefore, agreement in average transmission does not necessarily imply agreement in near-term escalation or operational burden.

Classical compartmental frameworks such as SIS, SIR, and their hospital-adapted variants have delivered valuable intuition by compressing complex biology into parsimonious transition rates (Cooper et al. 2004; Robotham et al. 2007; Chamchod and Ruan 2012; D’Agata et al. 2009; Weinstein et al. 2001). That compression becomes a limitation when biology imposes an internal clock. MRSA colonization is not a static state with uniform infectiousness. It is a dynamic process shaped by within-host replication, host immune engagement, and treatment, all of which evolve with **colonization age** (Bai 2023; Barril et al. 2023; Demongeot et al. 2023; Brauer 2005). Within-host trajectories can feature bacterial burden rising and saturating under intrinsic growth, followed by immune activation after a delay that suppresses burden. Under this view, the ward-level quantity often treated as a transmission rate is more naturally represented as a time-varying kernel driven by within-host burden and modulated by immune-mediated clearance (Childs et al. 2019; Gubbins 2024; Greenhalgh and Dumas 2023; Li et al. 2025). Constant-rate ward models can fit aggregate data and reproduce equilibria because they average over these trajectories. However, averaging removes the mechanistic timing that governs whether transmission is concentrated early in colonization, when patients are most likely still present and interacting, or shifted into later periods that many patients do not reach due to discharge or decolonization.

This motivates a structural phenomenon that constant-rate models cannot represent, front-loading. Front-loading occurs when a large fraction of total transmission potential accrues early in the colonization episode. When infectiousness is front-loaded, the timing of clearance and the distribution of infectiousness over colonization age can shape both stochastic ignition at low counts and cumulative burden after establishment (Wallinga and Lipsitch 2007; Wearing et al. 2005). We therefore ask the following question: If ICU MRSA transmissibility is colonization age dependent and front loaded by within-host kinetics, how do the resulting timing structure and mechanistically induced heterogeneity shape the probability that a single introduction establishes rather than dies out stochastically, and how do clearance timing and immune strength change cumulative ward burden in the fitted regime?

To address this question, we make infectiousness emergent and colonization-age dependent rather than assuming a constant transmission rate. At the within-host scale, bacterial burden and immune response trajectories define colonization-age kernels that determine the timing of transmissibility and progression risk. At the ward scale, we embed these colonization-age kernels in a colonization-age structured transmission model that tracks the distribution of colonized and infected patients by colonization age. Under this construction, the force of colonization at calendar time *t* depends on the current colonization-age densities, so transmission is governed by both intensity and timing relative to discharge, decolonization, and progression.

Because outbreak risk is decided at low counts, we pair the colonization-age structured ward dynamics with a stochastic ignition stage and then evaluate consequences in the fitted regime. Even when $R_0>1$, early spread begins with very small numbers of colonized patients, so chance events such as rapid discharge, early decolonization, or a short chain that fails to branch can terminate transmission before it becomes self-sustaining (Blumberg et al. 2014). We therefore represent ignition using a branching process whose mean and heterogeneity are grounded in the mechanistic model. We then simulate ward-scale dynamics conditional on establishment to quantify cumulative burden under variation in clearance timing and immune strength. Finally, we construct a constant-transmission ward comparator matched to the same $R_0$ as the colonization-age model. Under this matched-$R_0$ comparison, differences in establishment probability and cumulative burden are attributable to timing structure rather than differences in overall reproductive potential.

The remainder of this paper is organized as follows. Section 2 presents the methodology, coupling a within-host MRSA model to a colonization-age-structured ICU ward model. It also defines the basic reproduction number, constructs a matched-$R_0$ constant-transmission comparator, and describes the two-stage framework calibrated to Edmonton hospital data. Section 3 presents and discusses the results, focusing on stochastic establishment, ward burden under variation in clearance timing and immune strength, and the matched-$R_0$ comparison. Section 4 concludes by summarizing the main findings and their implications for ICU MRSA control. Appendix A provides supporting parameter derivations and literature-based justifications.

## Methodology

### Study Setting, Observables, and Modeling Targets

We study transmission of methicillin-resistant *Staphylococcus aureus* (MRSA) in an intensive care unit (ICU), where admissions, discharge and transfer, decolonization, and clinical progression jointly shape short-run outbreak dynamics. Our modeling objective is to quantify how colonization-age timing, induced by within-host kinetics, changes two decision-relevant outcomes: (i) the probability that a single introduction establishes rather than dies out stochastically, and (ii) the conditional ward burden once establishment occurs, summarized by cumulative patient-days and related burden measures over a fixed operational horizon. We further quantify how two within-host clearance levers, clearance timing and immune strength, change these outcomes in the fitted regime.

To isolate the role of timing structure, we construct two ward-scale models that share the same patient-flow backbone and differ only in their representation of transmissibility as a function of time since colonization. Model (i) is a colonization-age-dependent formulation in which within-host trajectories define a colonization-age-dependent transmission kernel $\beta (\tau )$ and a colonization-age-dependent progression mechanism. Model (ii) is a constant-transmission comparator in which transmissibility is time-invariant but calibrated to match the same basic reproduction number $R_0$ as Model (i). We pair both models with a two-stage framework in which Stage 1 describes stochastic ignition at low counts and returns the establishment probability, while Stage 2 simulates ward-scale dynamics conditional on establishment and returns burden summaries. Stage 1 also incorporates mechanistically induced heterogeneity through an effective dispersion parameter $k_{\textrm{disp}}$ computed from the individual-level transmission potential implied by the colonization-age model. Under this design, differences between Model (i) and Model (ii) can be attributed to colonization-age timing structure rather than differences in overall reproductive potential.

We construct the colonization-age mechanism by linking within-host MRSA bacterial burden and immune control to between-host transmission and progression. Specifically, we introduce colonization age τ as time since colonization and represent colonized and infected classes as colonization-age densities rather than single totals, so that patients with different τ contribute differently to onward spread and infection risk. Within-host kinetics generate trajectories for bacterial burden and host response, which together determine time-varying infectiousness through a transmissibility kernel $\beta (\tau )$ and a progression mechanism that depends on τ. Embedding these colonization-age mechanisms in an ICU ward model makes the force of colonization at calendar time *t* a functional of the current colonization-age distribution, thereby preserving timing structure that constant-rate formulations average away.

### Within-Ward Patient Dynamics: Baseline Flow Structure

We begin by specifying a shared ward-scale backbone that both models inherit and use an adult ICU with a nominal size of approximately 30 beds, consistent with prospective surveillance and intervention studies in infection control (Nseir et al. 2005; Ledoux et al. 2016; Rodríguez et al. 2025). The bed number provides clinical context rather than a hard capacity constraint in the differential equations. Patients are partitioned into four clinical states: susceptible (*S*), colonized on admission ($C_A$), colonized within the ward ($C_W$), and clinically infected (*I*). Patients enter at rate Λ patients per day. The effective importation parameter $\eta \in [0,1]$ allocates admissions between patients entering the colonized-on-admission class at rate $\eta \Lambda$ and patients entering the susceptible class at rate $(1-\eta )\Lambda$. We retain $C_A$ and $C_W$ as separate classes to distinguish effective external importation from within-ward acquisition, although both classes contribute to onward transmission.

The four ward compartments have the following meanings. *S*(*t*) is the number of patients who are currently MRSA-negative and therefore susceptible to colonization. $C_A(t)$ is the number of patients who entered the ICU already colonized with MRSA but are not currently in the clinical-infection state. $C_W(t)$ is the number of patients in the non-clinically-infected colonized reservoir generated through within-ward acquisition or through return from clinical infection after recovery. *I*(*t*) is the number of patients with clinical MRSA infection following progression from either colonized class. Thus, $C_A(t)$ distinguishes effective external importation, $C_W(t)$ represents the within-ward colonized reservoir, and *I*(*t*) includes clinical infection regardless of where the preceding colonization was acquired.

In the baseline compartmental representation, susceptible patients acquire MRSA colonization at rate λ(t)S. ICU surveillance studies such as Huskins et al. distinguish incident acquisition of MRSA colonization or infection and therefore support explicit representation of the within-ward acquisition pathway (Huskins et al. 2011). Colonized patients may subsequently progress to clinical infection. David and Daum describe the clinical spectrum of MRSA from asymptomatic carriage to severe infection, providing qualitative support for this colonization-to-infection transition (David and Daum 2010). These references justify the modeled biological pathways but do not provide a numerical value for the progression rate α. In the baseline equations, the transition is represented by α; in the colonization-age formulation, it is replaced by the burden-dependent hazard $\alpha (\tau )=\alpha _0B(\tau )$ defined in Eq. (8), with $\alpha _0$ estimated during calibration. Colonized patients may also be successfully decolonized at rate $r_C$, returning to susceptibility, or may leave the ICU at discharge and transfer rate μ. Infected patients exit through clinical recovery at rate γ, discharge and transfer at rate μ, or MRSA-attributable death at rate δ.

The baseline ICU dynamics can be written as:

1 $$\begin{aligned} \frac{dS}{dt}&= (1-\eta )\Lambda - \lambda (t)\, S - \mu S + r_C (C_A + C_W), \end{aligned}$$

2 $$\begin{aligned} \frac{dC_A}{dt}&= \eta \Lambda - (\alpha + \mu + r_C) C_A, \end{aligned}$$

3 $$\begin{aligned} \frac{dC_W}{dt}&= \lambda (t)\, S - (\alpha + \mu + r_C) C_W + \gamma I, \end{aligned}$$

4 $$\begin{aligned} \frac{dI}{dt}&= \alpha (C_A + C_W) - (\gamma + \mu + \delta ) I. \end{aligned}$$

Here, $r_C$ denotes successful decolonization (e.g., mupirocin therapy or chlorhexidine bathing), and we assume that clinical recovery resolves symptomatic infection but does not immediately clear carriage. Accordingly, recovered patients return to the colonized reservoir (represented by the +γI flow into $C_W$), while decolonization returns patients to susceptibility through the $r_C(C_A+C_W)$ term. The recovery rate γ is set using the mean duration of symptomatic MRSA infection reported by Hogea et al. (2014); an average duration of approximately 14.6 days corresponds to $\gamma = 1/14.6 \approx 0.069~\text {day}^{-1}$.

Equations (1)-(4) define the shared patient-flow backbone used in both the colonization-age model and the constant-transmission comparator. The key difference between models is how the acquisition force λ(t) and the progression hazard are constructed once infectiousness is allowed to vary with time since colonization.

The ward census generated by Eqs. (1)–(4) is governed by admissions, discharge or transfer, and infection-attributable mortality. Defining $N(t)=S(t)+C_A(t)+C_W(t)+I(t)$ and summing Eqs. (1)–(4) gives

$$ \frac{\textrm{d}N}{\textrm{d}t}=\Lambda -\mu N-\delta I. $$

At the disease-free equilibrium, $I^*=0$ and the equilibrium census is exactly $N^*=\Lambda /\mu$. At an equilibrium with clinical infection, $N^*=(\Lambda -\delta I^*)/\mu$ and is therefore slightly lower because of infection-attributable mortality. The baseline values Λ=3 patients per day and $\mu =0.1~\textrm{day}^{-1}$ imply a model-based disease-free equilibrium census of approximately 30 patients. This value represents a nominal near-capacity scenario for a 30-bed ICU; it is not an empirical estimate of the mean Edmonton ICU census, and the model does not impose 30 beds as a hard upper boundary. The aggregate surveillance series used for calibration does not contain a time-resolved ICU census or occupancy series from which the empirical mean census could be estimated. Using the 80–90% occupancy range identified by the reviewer, a 30-bed ICU would have an average census of approximately 24–27 patients. At the same discharge rate and in the absence of infection-attributable mortality, maintaining those mean census levels would require flow-balanced admission rates of approximately 2.4-−2.7 patients per day. These alternative occupancy levels were not imposed in the reported simulations, which should therefore be interpreted as representing the nominal near-capacity baseline defined by Λ=3 patients per day. In the final distributed-source analysis, the importation fraction is fixed at η=0.10, so the total colonized-admission rate is $\eta \Lambda =0.30$ patients per day. These admitted-colonized patients are not placed at colonization age zero; instead, they enter through the distributed source $\eta \Lambda g_A(\tau )$. Because $\int _0^\infty g_A(\tau )\,d\tau =1$, the distributed source preserves the total colonized-admission rate $\eta \Lambda$ while allocating admitted-colonized patients across pre-admission colonization ages. Sensitivity refits showed that the aggregate cumulative incidence series cannot identify η independently of the within-ward transmission parameters. Accordingly, η=0.10 is treated as a fixed modeling assumption and not as an estimated admission-screening prevalence.

### Within-Host MRSA Kinetics and Immune Response

Throughout the model, τ denotes the colonization-age coordinate used to index the within-host trajectories and the colonization-age-structured ward densities. For patients who acquire MRSA within the ICU, τ=0 is the actual time of within-ward acquisition, and these patients enter through the boundary $c_W(t,0)=S(t)\lambda (t)$. For patients who are already colonized at ICU admission, the true duration of pre-admission carriage is unobserved. These patients are therefore not concentrated at colonization age zero. Instead, they enter the admitted-colonized density through the distributed source $\eta \Lambda g_A(\tau )$ and are represented across $\tau \ge 0$ according to the normalized pre-admission colonization-age density $g_A(\tau )$ derived from the untreated colonized-state survival function $\pi _C^{(0)}(\tau )$. Thus, for admitted-colonized patients, τ represents total modeled colonization age, including an inferred pre-admission component, although the exact biological colonization age of any individual patient remains latent rather than directly observed. We describe the within-host dynamics using two state variables: B(τ), MRSA bacterial load at colonization age τ, and E(τ), host innate immune effector response at colonization age τ. In MRSA carriage and infection, a dominant protective mechanism is the innate immune response, particularly neutrophil-mediated killing, with contributions from macrophages and antimicrobial peptides (Rigby and Deleo 2012). We therefore interpret E(τ) as an aggregated measure of innate immune effector activity.

We model bacterial growth as logistic expansion tempered by immune-mediated killing and drug-mediated clearance, while immune effectors are produced basally and upregulated in response to bacterial burden:

5 $$\begin{aligned} \frac{dB(\tau )}{d\tau }&= r_{\text {bac}}\,B(\tau )\left( 1-\frac{B(\tau )}{K_{\max }}\right) -\kappa (\tau )\,B(\tau )\,E(\tau ) -\delta _{\text {drug}}\,B(\tau ), \end{aligned}$$

6 $$\begin{aligned} \frac{dE(\tau )}{d\tau }&= \sigma _{\text {imm}}+\omega \,B(\tau )-\delta _{\text {imm}}\,E(\tau ), \end{aligned}$$

with initial conditions $B(0)=B_0$ and $E(0)=E_{\text {base}}$. Here, $B_0=100~\textrm{CFU,ml}^{-1}$ denotes the assumed initial bacterial inoculum at colonization age τ=0, and $E_{\text {base}}$ denotes baseline innate immune activity. In (5), $r_{\text {bac}}$ is the intrinsic bacterial growth rate, $K_{\max }$ is the within-host carrying capacity, and $\delta _{\text {drug}}$ captures antibiotic- and treatment-related bacterial removal. The possibly time-varying immune killing rate is defined as $\kappa (\tau )=\kappa _{\max }/\left[ 1+\exp {-k_{\kappa }(\tau -\tau _{\textrm{crit}})}\right]$, where $\kappa _{\max }$ is the maximum immune killing coefficient, $\tau _{\textrm{crit}}$ is the onset time of effective clearance, and $k_{\kappa }$ controls the steepness of the transition. The biological motivation and parameterization of this function are provided in Appendix A under “Immune killing-rate transition ($\kappa (\tau )$).” In (6), $\sigma _{\text {imm}}$ is a basal source of immune effectors, ω captures immune stimulation by bacterial burden, and $\delta _{\text {imm}}$ is effector decay. This within-host system provides the mechanistic basis for colonization-age dependence. In particular, rapid early growth in B(τ) can generate a period of elevated shedding potential before immune- or treatment-mediated clearance becomes effective, which is the timing structure evaluated against establishment probability and cumulative ward burden in later sections.

### Coupling to Epidemiology: Colonization-Age Transmission and Progression Kernels

We translate within-host bacterial burden into ward-scale hazards by allowing transmission and clinical progression to depend on B(τ). We assume that the effective transmission contribution of an individual with colonization age τ increases with bacterial load but saturates due to biological limits on shedding and contact processes. We represent this relationship using a Hill (saturating) function:

7 $$\begin{aligned} \beta (\tau )\equiv \beta \!\left( B(\tau )\right) = \beta _{\max }\,\frac{B(\tau )^{h}}{B(\tau )^{h}+B_{50}^{h}}, \end{aligned}$$

where $\beta _{\max }$ is the maximum effective transmissibility contribution, $B_{50}$ is the bacterial load at which transmissibility reaches half its maximum, and *h* is the Hill coefficient controlling steepness. Under the frequency-dependent force of colonization in (17), $\beta (\tau )$ has units of $\text {day}^{-1}$ and represents an aggregated effective contact times transmission rate contributed by an individual at colonization age τ.

We also allow the instantaneous rate at which colonization progresses to clinical infection to increase with bacterial burden:

8 $$\begin{aligned} \alpha (\tau )\equiv \alpha \!\left( B(\tau )\right) =\alpha _{0}\,B(\tau ), \end{aligned}$$

where $\alpha _{0}$ scales how bacterial load translates into infection risk. Equations (7) and (8) convert within-host kinetics into colonization-age dependent transmission and progression kernels.

The two kernels create the timing structure that is evaluated in the study endpoints. First, $\beta (\tau )$ shapes when secondary colonizations occur and therefore affects the establishment probability in Stage 1 through the mean $R_0$ and the effective dispersion parameter $k_{\textrm{disp}}$ derived from individual transmission potential. Second, $\alpha (\tau )$ shapes when individuals move into infection and when they leave transmissible states through recovery or mortality, which in turn changes the cumulative ward burden in Stage 2 under different clearance timing and immune strength settings.

### Ward Dynamics with Colonization-Age Structure

We embed the colonization-age dependent kernels $\beta (\tau )$ and $\alpha (\tau )$ into the shared ICU flow backbone by representing colonized and infected patients as colonization-age densities. Let $c_A(t,\tau )$ denote the density of patients colonized on admission at time *t* with colonization age τ, $c_W(t,\tau )$ the density of patients colonized within-ward with colonization age τ, and i(t,τ) the density of clinically infected patients indexed by the same model colonization age τ (as defined in Section 2.3). The corresponding totals are obtained by integrating over colonization age:

$$ C_A(t)=\int _{0}^{\infty } c_A(t,\tau )\,d\tau ,\qquad C_W(t)=\int _{0}^{\infty } c_W(t,\tau )\,d\tau ,\qquad I(t)=\int _{0}^{\infty } i(t,\tau )\,d\tau . $$

The ICU census is

$$ N(t)=S(t)+\int _{0}^{\infty }\!\big (c_A(t,\tau )+c_W(t,\tau )+i(t,\tau )\big )\,d\tau , $$

and we define the total colonized-or-infected burden

$$ P(t)=\int _{0}^{\infty }\!\big (c_A(t,\tau )+c_W(t,\tau )+i(t,\tau )\big )\,d\tau . $$

Governing equations. Susceptible patients are not colonization-aged, so *S*(*t*) remains an ODE variable. To represent colonized admissions with heterogeneous pre-admission carriage durations, define

9 $$\begin{aligned} g_A(\tau )=\frac{\pi _C^{(0)}(\tau )}{\displaystyle \int _0^\infty \pi _C^{(0)}(\sigma )\,d\sigma }, \end{aligned}$$

where $\pi _C^{(0)}(\tau )$ is obtained from the single-individual survival system in Eqs. (19)–(20) after setting $\delta _{\textrm{drug}}=0$ in the within-host bacterial equation. The function $g_A(\tau )$ is normalized so that $\int _0^\infty g_A(\tau )\,d\tau =1$ and therefore describes the assumed colonization-age distribution of patients admitted while colonized. The colonization-age-structured ward model is:

10 $$\begin{aligned} \frac{dS(t)}{dt} &  = (1-\eta )\Lambda -\lambda (t)S(t)-\mu S(t)+\int _0^\infty r_C(\tau )\big [c_A(t,\tau )\nonumber \\ &  \quad \ +c_W(t,\tau )\big ]\,d\tau , \end{aligned}$$

11 $$\begin{aligned} \frac{\partial c_A(t,\tau )}{\partial t}+\frac{\partial c_A(t,\tau )}{\partial \tau } &  = -\big (\alpha (\tau )+\mu +r_C(\tau )\big )c_A(t,\tau )+{\eta \Lambda g_A(\tau )}, \end{aligned}$$

12 $$\begin{aligned} \frac{\partial c_W(t,\tau )}{\partial t}+\frac{\partial c_W(t,\tau )}{\partial \tau } &  = -\big (\alpha (\tau )+\mu +r_C(\tau )\big )c_W(t,\tau )+\gamma (\tau )i(t,\tau ), \end{aligned}$$

13 $$\begin{aligned} \frac{\partial i(t,\tau )}{\partial t}+\frac{\partial i(t,\tau )}{\partial \tau } &  = \alpha (\tau )\big [c_A(t,\tau )+c_W(t,\tau )\big ]\nonumber \\ &  \quad \ -\big (\gamma (\tau )+\mu +\delta \big )i(t,\tau ). \end{aligned}$$

The distributed source $\eta \Lambda g_A(\tau )$ in Eq. (11) admits colonized patients across colonization ages rather than placing every colonized admission at age zero. Because $g_A$ integrates to one, integrating this source over all colonization ages gives the original total colonized-admission rate $\eta \Lambda$. Here, $\alpha (\tau )$ is the burden-driven progression kernel from (8), and $\beta (\tau )$ is the burden-driven transmissibility kernel from (7). We allow decolonization $r_C(\tau )$ and recovery $\gamma (\tau )$ to be colonization-age-dependent, while retaining discharge and transfer μ and infection-attributable mortality δ as constant hazards. Setting $r_C(\tau )\equiv r_C$ and $\gamma (\tau )\equiv \gamma$ recovers the constant-hazard backbone in Sect. 2.2.

**Boundary conditions:** importation and within-ward acquisition. Because colonized admissions now enter through the distributed source $\eta \Lambda g_A(\tau )$ in Eq. (11), no admitted-colonized patients enter through the age-zero boundary. Consequently,

14 $$\begin{aligned} \boldsymbol{c_A(t,0)=0}. \end{aligned}$$

New within-ward acquisitions occur at colonization age zero, so

15 $$\begin{aligned} c_W(t,0)=S(t)\lambda (t). \end{aligned}$$

Clinical infections arise only through progression from colonization within the model, and therefore

16 $$\begin{aligned} i(t,0)=0. \end{aligned}$$

This formulation distinguishes the unknown pre-admission carriage durations of colonized admissions from genuine within-ward acquisitions, whose colonization-age clocks begin at the time of acquisition. The density $g_A(\tau )$ follows the reviewer-suggested survival-based approximation with drug-mediated bacterial clearance omitted before admission.

**Force of colonization under colonization-age dependent transmissibility.** Because individual infectiousness depends on colonization age, the acquisition hazard acting on susceptibles aggregates transmissibility across the entire colonization-age distribution:

17 $$\begin{aligned} \lambda (t)=\frac{1}{N(t)}\int _{0}^{\infty }\beta (\tau )\, \Big [c_A(t,\tau )+c_W(t,\tau )+i(t,\tau )\Big ]\,d\tau , \end{aligned}$$

where $\beta (\tau )=\beta (B(\tau ))$ is defined by (7). Equation (17) is the mechanism through which within-host timing propagates to ward acquisition and, through the evolving burden *P*(*t*), to cumulative patient-days in Stage 2. In the next subsection, we define $R_0$ in a way that is consistent with colonization-age structure and ICU turnover, which is needed for the Stage 1 branching approximation and for constructing the constant-transmission comparator used in the timing-only comparison.

### Computing $R_0$ and the Front-Loading Diagnostic

The matched-$R_0$ counterfactual and the Stage 1 branching approximation require a definition of $R_0$ that correctly accounts for time-varying infectiousness and for removal from transmissible states through discharge and transfer, decolonization, progression to infection, recovery, and mortality. In the colonization-age model, a newly colonized patient does not transmit at a constant rate. Instead, transmissibility evolves with colonization age through $\beta (\tau )$, while occupancy of colonized and infected states evolves through $\alpha (\tau )$ and $\gamma (\tau )$. We therefore compute $R_0$ as the expected cumulative number of secondary ward-acquired colonizations generated by a single newly colonized individual over their full colonization-age trajectory in an otherwise susceptible ward.

**Disease-free susceptible pool and cohort representation.** Because transmission is frequency-dependent, the instantaneous production of new colonizations includes the susceptible fraction *S*(*t*)/*N*(*t*). To recover the standard $R_0$ interpretation as the expected number of secondary cases generated in a fully susceptible environment, we evaluate this factor at the theoretical disease-free equilibrium (DFE).

A true DFE requires the complete absence of the pathogen, meaning that there is no within-ward acquisition, λ(t)=0, and no external importation of colonized patients, η=0. Under these theoretical conditions, the differential equation for susceptible patients reduces to

18 $$\begin{aligned} \frac{dS}{dt}=\Lambda -\mu S \quad \Longrightarrow \quad S^*=\frac{\Lambda }{\mu }. \end{aligned}$$

Because no other clinical states are populated, the total steady-state census is $N^*=\Lambda /\mu$. Therefore, at the DFE, the susceptible fraction is exactly $S^*/N^*=1$. We use this standard normalization for frequency-dependent transmission when computing the expected number of secondary ward acquisitions produced by a single newly colonized introduction. In the fitted operational simulations, η>0 is retained to represent the continuing external seeding of the ICU by colonized admissions; under those conditions, a true disease-free equilibrium does not exist. Accordingly, the reported $R_0$ measures intrinsic within-ward reproductive potential in the theoretical importation-free environment and does not count imported colonized admissions as secondary cases.

**Single-individual survival in transmissible states.** Consider a single newly colonized individual introduced at colonization age τ=0. Along the colonization-age characteristic t=τ, we define $\pi _C(\tau )$ as the probability that the individual is in the colonized state at colonization age τ, whether the individual has remained continuously in that state or has returned to it after recovery from infection. Similarly, $\pi _I(\tau )$ is the probability that the individual is in the infected state at colonization age τ after progression from colonization. These state-occupancy probabilities summarize the time that a typical introduced individual spends in transmissible states and therefore determine how colonization-age timing shapes both cumulative transmission potential and the Stage 1 finite-threshold probability. They satisfy

19 $$\begin{aligned} \frac{d\pi _C}{d\tau }&= -\bigl (\alpha (\tau )+\mu +r_C(\tau )\bigr )\pi _C(\tau ) +\gamma (\tau )\pi _I(\tau ), \end{aligned}$$

20 $$\begin{aligned} \frac{d\pi _I}{d\tau }&= \alpha (\tau )\pi _C(\tau ) -\bigl (\gamma (\tau )+\mu +\delta \bigr )\pi _I(\tau ). \end{aligned}$$

With initial conditions $\pi _C(0)=1$ and $\pi _I(0)=0$, the quantity $\pi _C(\tau )+\pi _I(\tau )$ is the probability that the individual is present in either transmissible clinical state, colonized or infected, at colonization age τ, after accounting for ICU exit, decolonization, progression, recovery, and mortality. The gain term $\gamma (\tau )\pi _I(\tau )$ in Eq. (19) represents return from the infected state to the colonized state following recovery. Thus, $\pi _C(\tau )$ is a current state-occupancy probability and does not require the individual to have remained continuously in the colonized compartment.

${\textbf {R}}_{{\textbf {0}}}$ **as a next-generation integral.** The next-generation integral used here follows the general age-of-infection framework described by Yang and Brauer (2008); our contribution is to specialize that established construction to the present colonization-age-structured MRSA model. Because colonization age is continuous, the appropriate next-generation object is an integral operator rather than a standard finite-dimensional next-generation matrix. In the present model, this operator maps one newly colonized individual entering at τ=0 to the expected production of newly colonized secondary cases. Because every secondary ward acquisition enters the same colonized state at τ=0, the model has a single birth type, and the spectral radius of the next-generation operator reduces to the scalar integral given below. Under frequency-dependent transmission, the instantaneous rate at which a single introduced individual generates new ward acquisitions at colonization age τ is

$$\begin{aligned} R_{\textrm{new}}(\tau ) = \beta (\tau ) \bigl (\pi _C(\tau )+\pi _I(\tau )\bigr ) \frac{S^*}{N^*}. \end{aligned}$$

Because $S^*/N^*=1$ exactly at the theoretical DFE, we obtain the next-generation integral representation

21 $$\begin{aligned} R^{\textrm{age}}_0 = \int _0^\infty R_{\textrm{new}}(\tau )\,d\tau = \int _0^\infty \beta (\tau ) \bigl (\pi _C(\tau )+\pi _I(\tau )\bigr )\,d\tau . \end{aligned}$$

Here, $\beta (\tau )$ is defined by (7) through B(τ), while $\alpha (\tau )$ is defined by (8). Equation (21) is the specialization of the established next-generation integral framework to the present mechanistic model and accounts for when transmission occurs relative to ICU turnover and clinical progression.

To compute $R^{\textrm{age}}_0$ without applying a separate post-processing quadrature rule to Eq. (21), we introduce the cumulative reproduction number as an additional state variable,

$$\begin{aligned} R_0(\tau )=\int _0^\tau R_{\textrm{new}}(\sigma )\,d\sigma . \end{aligned}$$

By the fundamental theorem of calculus, this state variable satisfies

$$\begin{aligned} \frac{dR_0(\tau )}{d\tau }=R_{\textrm{new}}(\tau ), \qquad R_0(0)=0. \end{aligned}$$

We append this equation to the within-host equations (5)–(6) and the cohort state-occupancy equations (19)–(20), and solve the augmented system for $\bigl (B(\tau ),E(\tau ),\pi _C(\tau ),\pi _I(\tau ),R_0(\tau )\bigr )$ over $0\le \tau \le T_{\textrm{end}}$. The terminal colonization age $T_{\textrm{end}}$ is chosen sufficiently large that $\pi _C(T_{\textrm{end}})+\pi _I(T_{\textrm{end}})$ is negligible and the cumulative state $R_0(\tau )$ has stabilized. Consequently,

$$\begin{aligned} R^{\textrm{age}}_0 = \lim _{\tau \rightarrow \infty }R_0(\tau ) \approx R_0(T_{\textrm{end}}). \end{aligned}$$

Thus, the reported value of $R^{\textrm{age}}_0$ is obtained directly as the terminal value of the augmented ODE state rather than through a separate numerical quadrature of the next-generation integral.

**A quantitative diagnostic for front-loading.** To measure timing structure directly, we define the $R_0$ integrand

22 $$\begin{aligned} g(\tau ) \equiv \beta (\tau )\,\big (\pi _C(\tau )+\pi _I(\tau )\big ), \end{aligned}$$

so that $R_0^{\textrm{age}}=\int _0^\infty g(\tau )\,d\tau$. We then define the cumulative transmission share up to colonization age *t* (with *t* measured in days) as

23 $$\begin{aligned} F(t) \equiv \frac{\int _{0}^{t} g(\tau )\,d\tau }{\int _{0}^{\infty } g(\tau )\,d\tau }. \end{aligned}$$

The function *F*(*t*) provides a direct, model-based measure of front-loading. For example, $F(7\,\textrm{days})$ is the fraction of total transmission potential realized within the first week of carriage. This timing diagnostic is later used to interpret why changes in clearance timing and immune strength can alter cumulative ward burden even when $R_0$ is held fixed in the matched-$R_0$ comparison.

### Constructing the Constant-Rate Comparator by Matching $R_0$

We formalize the counterfactual constant-rate ward model used for the matched-$R_0$ comparison. The purpose of this comparator is not to represent a different biology, but to test the operational implication of colonization-age timing: whether preserving long-run reproductive potential ($R_0$) is sufficient for predicting establishment risk and cumulative ward burden when transmissibility is strongly time-varying. Accordingly, the comparator preserves the same patient-flow backbone (admission, discharge and transfer, decolonization, progression, recovery, and mortality) and differs only in the timing structure of transmissibility.

**Definition of the constant-rate transmissibility kernel.** In the comparator, we replace the colonization-age dependent kernel $\beta (\tau )=\beta (B(\tau ))$ by a time-invariant effective value,

24 $$\begin{aligned} \beta _{\textrm{const}}(\tau )\equiv \bar{\beta }. \end{aligned}$$

All other ward-scale mechanisms are kept identical to the colonization-age formulation, including the same μ, δ, and the same functional forms for $r_C(\tau )$ and $\gamma (\tau )$ when age-dependence is used.

**Matching constraint:** $R_0^{\textrm{const}}=R_0^{\textrm{age}}$ **.** Because the comparator has constant transmissibility, its reproduction number is

25 $$\begin{aligned} R_0^{\textrm{const}} = \int _{0}^{\infty } \bar{\beta }\,\big (\pi _C(\tau )+\pi _I(\tau )\big )\,d\tau = \bar{\beta }\,A, \qquad A \equiv \int _{0}^{\infty }\big (\pi _C(\tau )+\pi _I(\tau )\big )\,d\tau , \end{aligned}$$

where $\pi _C(\tau )$ and $\pi _I(\tau )$ are defined by (19) and (20). We choose $\bar{\beta }$ so that the comparator matches the colonization-age model:

26 $$\begin{aligned} R_0^{\textrm{const}} = R_0^{\textrm{age}}. \end{aligned}$$

Combining (21) and (25) yields the explicit matching rule

27 $$\begin{aligned} \bar{\beta } = \frac{R_0^{\textrm{age}}}{\int _{0}^{\infty }\big (\pi _C(\tau )+\pi _I(\tau )\big )\,d\tau }. \end{aligned}$$

This choice preserves total transmission potential while removing colonization-age variation.

**Timing-only contrast.** Under this construction, the $R_0$ integrand for the constant-rate comparator is

28 $$\begin{aligned} g_{\textrm{const}}(\tau ) \equiv \bar{\beta }\,\big (\pi _C(\tau )+\pi _I(\tau )\big ), \end{aligned}$$

while the colonization-age model integrand is g(τ) from (22). By design,

$$ \int _0^\infty g_{\textrm{const}}(\tau )\,d\tau = \int _0^\infty g(\tau )\,d\tau , $$

so both models share the same $R_0$ even though the timing profile can differ substantially. This defines a timing-only contrast. Any differences in early acceleration, trajectory shape, or cumulative patient-days over a fixed operational horizon arise from when transmission occurs, not from how much transmission occurs in total. At this point, the two ward models needed for the matched-$R_0$ comparison are fully defined.

### Two-Stage Stochastic-Hybrid Simulation Framework

The central question in this work is not only how much transmission is possible, as summarized by $R_0$, but also when that transmission occurs within a patient. When transmissibility is front-loaded and depends on colonization age, a constant-transmission ward model that is matched to the same $R_0$ can preserve long-run reproductive potential while changing the timing of transmission events. This matters for two endpoints that match our research question. First, we quantify whether a single introduction establishes or dies out by chance when transmissibility is colonization-age dependent and heterogeneity is induced by the coupled mechanisms. Second, conditional on establishment, we quantify how clearance timing and immune strength change cumulative ward burden in the fitted regime. We also use a matched-$R_0$ constant-transmission comparator to isolate the role of timing structure in these operational outcomes.

To address these endpoints, we use a two-stage pipeline. Stage 1 represents low-number ignition as a stochastic process and returns the probability of establishment versus extinction. Stage 2 simulates ward-scale dynamics conditional on establishment and returns cumulative burden summaries under the colonization-age structured model. Stage 2 is also simulated under a constant-transmission comparator calibrated to the same $R_0$.

Because the nominal ward census is small, demographic stochasticity may remain important even after the process reaches the operational handoff threshold $Z_{\textrm{crit}}$. The hybrid framework addresses only part of this finite-population issue. Stage 1 uses a stochastic branching process to represent extinction and threshold crossing when case numbers are low, whereas Stage 2 uses the deterministic ward equations, initialized at the handoff state, to compare post-handoff trajectories and cumulative burden. The Stage 2 solution is therefore a conditional mean-field approximation; it is not the exact expectation of a finite-population stochastic model and does not represent the full distribution of post-handoff outcomes, ongoing demographic fluctuations, or dependence induced by a hard 30-bed capacity. A fully stochastic individual-based implementation that repeatedly solves the coupled within-host dynamics for every patient would better represent these features, but would be computationally prohibitive within the $10^6$-sample calibration and the subsequent paired simulation experiments conducted here. Accordingly, the reported Stage 2 outcomes should be interpreted as deterministic, model-implied comparisons of conditional ward burden rather than exact stochastic forecasts for a 30-patient ICU. Extending the framework to a capacity-constrained, finite-population stochastic ward model is an important direction for future work.

**Stage 1: stochastic establishment using a branching process.** Stage 1 represents the introduction phase, when only a few patients are colonized and outcomes are dominated by chance. We approximate early spread using a discrete-time Galton-Watson branching process. Let $Z_n$ denote the number of newly colonized patients in generation *n*, with

$$ Z_0=1. $$

Each colonized patient in generation *n* independently generates a random number of secondary colonizations in generation n+1, so

29 $$\begin{aligned} Z_{n+1}=\sum _{i=1}^{Z_n} Y_{n,i}, \end{aligned}$$

where the offspring counts $\{Y_{n,i}\}$ are i.i.d. nonnegative integer-valued random variables. We parameterize the offspring distribution so that

$$ \mathbb {E}[Y]=R_0, $$

matching the mean reproductive potential implied by the mechanistic ward model.

To represent heterogeneity during ignition, we use an effective negative binomial offspring distribution with mean $R_0$ and dispersion parameter $k_{\textrm{disp}}>0$, parameterized so that

$$ \textrm{E}[Y]=R_0, \qquad \textrm{Var}(Y)=R_0+\frac{R_0^2}{k_{\textrm{disp}}}, $$

where *Y* is the integer-valued number of secondary ward-acquired colonizations generated by one introduced colonized individual.

In this work, $k_{\textrm{disp}}$ is not treated as a free parameter fitted to the Edmonton incidence series. Instead, it is derived from between-individual variation in cumulative transmission opportunity generated by the mechanistic colonization-age model. Let *T* denote the random first-exit time at which an introduced individual leaves the union of the transmissible colonized and infected states through discharge or transfer, decolonization, or mortality. Progression from colonization to infection and recovery from infection to colonization do not end the transmission episode because both states remain transmissible. The survival function and distribution function of *T* are therefore

$$ S_T(\tau )=\Pr (T>\tau )=\pi _C(\tau )+\pi _I(\tau ) $$

and

$$ F_T(\tau )=\Pr (T\le \tau )=1-S_T(\tau )=1-\bigl (\pi _C(\tau )+\pi _I(\tau )\bigr ). $$

For a randomly selected introduced individual, define the random cumulative transmission potential

$$ V=\int _0^T\beta (\tau )\,d\tau . $$

The uppercase symbol *V* denotes a random variable because the exit time *T* varies among individuals. For one individual whose realized exit time is $T_i$, the corresponding realized transmission potential is the fixed value

$$ v_i=\int _0^{T_i}\beta (\tau )\,d\tau . $$

The expectation of *V* satisfies

$$ \textrm{E}[V] = \int _0^\infty \beta (\tau )\Pr (T>\tau )\,d\tau = \int _0^\infty \beta (\tau )\bigl (\pi _C(\tau )+\pi _I(\tau )\bigr )\,d\tau = R_0^{\textrm{age}} \equiv R_0. $$

Virtual individuals are generated from this mechanistic exit-time distribution by inverse-transform sampling. We first solve the within-host equations for B(τ) and E(τ) and the state-occupancy equations for $\pi _C(\tau )$ and $\pi _I(\tau )$ on the common colonization-age grid. These solutions determine $\beta (\tau )$ and $F_T(\tau )$. For virtual individual *i*, we draw

$$ U_i\sim \textrm{Uniform}(0,1), $$

set

$$ T_i=F_T^{-1}(U_i) = \inf \left\{ \tau :F_T(\tau )\ge U_i\right\} , $$

and calculate

$$ v_i=\int _0^{T_i}\beta (\tau )\,d\tau . $$

Thus, a virtual-individual trajectory in this calculation consists of a mechanistically sampled exit time $T_i$ and the transmission opportunity accumulated along $\beta (\tau )$ up to that exit time. The effects of progression and recovery are already incorporated into the exit-time distribution through $\pi _C(\tau )$ and $\pi _I(\tau )$; they are not separately refitted for each virtual individual.

Conditional on an individual having the realized transmission potential V=v, we assume

$$ Y\mid V=v\sim \operatorname {Poisson}(v), $$

where *Y* is the integer-valued number of secondary ward-acquired colonizations generated by that individual. This statement means that after the value *v* is fixed for a particular individual, the conditional Poisson mean is *v*. It does not mean that *V* is a fixed parameter across all individuals. Unconditionally, *Y* has a mixed-Poisson distribution obtained by averaging over the distribution of the random variable *V*:

$$ \Pr (Y=y) = \textrm{E}\!\left[ \frac{e^{-V}V^y}{y!} \right] , \qquad y=0,1,2,\ldots . $$

The laws of total expectation and total variance give

$$ \textrm{E}[Y]=\textrm{E}[V]=R_0 $$

and

$$ \textrm{Var}(Y) = \textrm{E}\!\left[ \textrm{Var}(Y\mid V)\right] + \textrm{Var}\!\left( \textrm{E}[Y\mid V]\right) = \textrm{E}[V]+\textrm{Var}(V) = R_0+\textrm{Var}(V). $$

We represent this mixed-Poisson offspring distribution by an effective negative binomial distribution having the same mean and variance. Equating its variance to $R_0+R_0^2/k_{\textrm{disp}}$ yields

$$ k_{\textrm{disp}} = \frac{R_0^2}{\textrm{Var}(V)}. $$

Accordingly, greater between-individual variation in cumulative transmission potential produces a smaller effective dispersion parameter and greater offspring heterogeneity. This moment-matching construction does not assume that *V* itself follows a negative binomial distribution. Instead, it provides an effective negative binomial representation of the mechanistically generated offspring-count distribution and ensures that Stage 1 incorporates the same colonization-age-dependent transmissibility and removal mechanisms that drive Stage 2.

**Stopping rule and establishment threshold.** We iterate (29) generation by generation until either extinction occurs ($Z_n=0$), establishment occurs ($Z_n\ge Z_{\textrm{crit}}$), or a safety cap $n=n_{\max }$ is reached. In this study we take

$$ Z_{\textrm{crit}}=5, $$

so an introduction is labeled established once a generation reaches at least five newly colonized patients.

**Stage 2 handoff (conditional ward simulation).** For realizations that establish, we initialize the ward model at time $t_0$ by seeding the unit with $Z_{\textrm{crit}}$ hospital-acquired colonized patients and reducing the susceptible count accordingly:

$$ C_W(t_0)=Z_{\textrm{crit}}, \qquad S(t_0)=N_0-Z_{\textrm{crit}}. $$

We then simulate the full ward-scale dynamics conditional on establishment to quantify cumulative ward burden under the colonization-age structured model. We repeat the same conditional simulation under the matched-$R_0$ constant-transmission comparator. In the fitted regime, we then evaluate how varying clearance timing (through $\tau _{\textrm{crit}}$ in the within-host clearance schedule) and immune strength (through $\kappa _{\max }$) changes cumulative patient-days and related burden summaries.

### Variables, Units, and Parameterization

We list the state variables and parameters used across the coupled within-host and ward-scale model and record units and nominal ranges.

**State variables and units.** Table 1 summarizes the main state variables and their units in the coupled within-host and ward-scale framework. For Stage 2 burden summaries we use *P*(*t*) for the total colonized-or-infected burden.

**Table 1:** Description and units of state variables in the coupled within-host and ward-scale MRSA model

Variable	Description	Units
*S*(*t*)	Susceptible (MRSA-negative) patients in the ICU	Patients
$c_A(t,\tau )$	Density of patients colonized on admission with colonization age τ	Patients/day
$c_W(t,\tau )$	Density of patients colonized within-ward with colonization age τ	Patients/day
i(t,τ)	Density of clinically infected patients with colonization age τ	Patients/day
$C_A(t)$	Total admitted-colonized patients	Patients
$C_W(t)$	Total ward-colonized patients	Patients
*I*(*t*)	Total clinically infected patients	Patients
*N*(*t*)	Total ICU census	Patients
*P*(*t*)	Total colonized-or-infected burden, $P(t)=C_A(t)+C_W(t)+I(t)$	Patients
λ(t)	Force of colonization (per-susceptible acquisition hazard)	$\text {day}^{-1}$
B(τ)	Within-host MRSA bacterial load at colonization age τ	CFU/ml
E(τ)	Innate immune effector activity at colonization age τ	Cells/ml

**Parameter descriptions and nominal ranges.** Table 2 lists within-host parameters, coupling parameters, and ward-scale parameters, along with nominal values and plausible ranges used for calibration and scenario exploration. Within-host sources commonly report rates per hour. When constructing colonization-age kernels for the ward model, we evaluate the within-host system on an hourly grid and convert time to days so that all ward-scale hazards are expressed in $\text {day}^{-1}$.

**Table 2:** Key parameters for the coupled within-host and ward-scale MRSA model. Within-host rates are parameterized on an hourly scale and mapped to the ward model, whereas ward-scale rates and hazards are expressed per day.

Symbol	Description	Value/Range	Reference
Ward-scale patient-flow parameters (day scale)			
Λ	Total ICU admission rate	3 patients $\text {day}^{-1}$	Assumed
μ	Discharge or transfer rate	$0.1~\textrm{day}^{-1}$	Assumed
Within-host parameters (hour scale unless otherwise stated)			
$B_0$	Initial bacterial inoculum at colonization age τ=0	$100~\mathrm {CFU\,ml}^{-1}$	Assumed
$r_{\text {bac}}$	Intrinsic bacterial growth rate	$1.386~\textrm{hr}^{-1}$	Pray (2008)
$K_{\max }$	Bacterial carrying capacity	$10^{10}~\mathrm {CFU\,ml}^{-1}$	Ridder et al. (2022)
$\kappa _{\max }$	Maximum immune killing coefficient	$6.4\times 10^{-6}~\mathrm {ml\,hr}^{-1}\textrm{cell}^{-1}$	Ridder et al. (2022)
$\tau _{\text {crit}}$	Onset time of effective clearance	9 days (baseline); varied from 5 to 15 days	Ridder et al. (2022)
$k_{\kappa }$	Steepness of the clearance transition	0.05 to $0.1~\textrm{hr}^{-1}$	Ridder et al. (2022)
$\delta _{\text {drug}}$	Drug-mediated bacterial clearance rate	0.033 to $0.069~\textrm{hr}^{-1}$	Pujol et al. (2021)
$\delta _{\text {imm}}$	Immune effector decay rate	$1~\textrm{hr}^{-1}$	Pugliese and Gandolfi (2008)
$\sigma _{\text {imm}}$	Basal immune effector source rate	$0.05\,\delta _{\text {imm}}E_{\text {base}}$	Pugliese and Gandolfi (2008)
ω	Immune stimulation by bacterial burden	0.02 to 0.05	Pugliese and Gandolfi (2008)
Calibrated coupling parameters and fixed admission parameter			
$\beta _{\max }$	Maximum effective transmissibility contribution	Median: $0.2263~\textrm{day}^{-1}$; range: $[0.1710,\,0.2601]~\textrm{day}^{-1}$	Final refit
$B_{50}$	Bacterial load at half-maximum transmissibility	Median: $5.22\times 10^{7}~\mathrm {CFU\,ml}^{-1}$; range: $[3.11\times 10^{6},\,9.76\times 10^{7}]~\mathrm {CFU\,ml}^{-1}$	Final refit
$\alpha _0$	Scaling coefficient for the progression hazard $\alpha (\tau )$	Median: $4.98\times 10^{-8}~\mathrm {ml\,CFU}^{-1}\textrm{day}^{-1}$; range: $[2.47\times 10^{-9},\,9.75\times 10^{-8}]~\mathrm {ml\,CFU}^{-1}\textrm{day}^{-1}$	Final refit
η	Fraction of admissions that are colonized	0.10 (fixed; not fitted)	Fixed
Ward-scale decolonization (day scale)			
$r_C(\tau )$	Decolonization hazard comprising natural clearance and a waning intervention contribution	$r_C(\tau )=0.0027+0.0075e^{-\rho \tau }~\textrm{day}^{-1}$; see Appendix A	Bootsma et al. (2006); Huskins et al. (2011)

For each sampled coupling parameter, the reported value is the behavioral median with the 95% behavioral range, defined by the 2.5th and 97.5th percentiles across the 45, 250 parameter sets satisfying cumulative $R^2\ge 0.90$. The importation fraction η=0.10 was fixed in the final distributed-source refit and was not sampled

Derivations and literature-based justifications for selected within-host and coupling parameters are provided in Appendix A

### Parameter Intervals for Latin Hypercube Sampling (LHS)

The final distributed-source calibration sampled three coupling parameters, $\beta _{\max }$, $B_{50}$, and $\alpha _0$, using a three-dimensional Latin Hypercube Sampling design over the candidate-search intervals reported in Table 3. The importation fraction was fixed at η=0.10 in every final calibration run and was therefore not included in the LHS design. The intervals in Table 3 are prespecified parameter-search bounds and should not be interpreted as confidence intervals, credible intervals, posterior intervals, or accepted behavioral ranges.

**Table 3:** Parameter-search intervals and fixed importation setting used in the final distributed-source calibration.

Parameter	Interpretation	Final calibration setting	Basis
$\beta _{\max }$	Maximum effective transmissibility contribution	$[0.1,\,1.0]~\textrm{day}^{-1}$	Broad search range
$B_{50}$	Bacterial load at half-maximum transmissibility	$[10^{5},\,10^{8}]~\mathrm {CFU\,ml}^{-1}$	Assumed range
$\alpha _0$	Scaling coefficient linking bacterial burden to the progression hazard $\alpha (\tau )$	$[10^{-12},\,10^{-7}]$ $\mathrm {ml\,CFU}^{-1}\textrm{day}^{-1}$	Assumed range
η	Fraction of admissions that are colonized	0.10 (fixed; not sampled)	Fixed after sensitivity analysis

The LHS sampled $\beta _{\max }$, $B_{50}$, and $\alpha _0$, whereas η was fixed at 0.10 and was not sampled

### Calibration and Uncertainty Propagation

Data and calibration target (Edmonton hospital-acquired MRSA). Model calibration used an aggregate, de-identified monthly time series of newly identified hospital-acquired MRSA colonization from Edmonton-area acute care and acute tertiary rehabilitation facilities, spanning September 2018 through August 2023. The dataset was obtained from the provincial surveillance data management system within the Alberta Health Services (AHS) Infection Prevention and Control (IPC) program, which records incident MRSA colonizations and infections and classifies cases by acquisition category (Alberta Health Services 2022, 2024a). Under the IPC definition, a case is classified as hospital-acquired when MRSA is first detected after the third calendar day of admission, with the admission day counted as day 1 (Alberta Health Services 2022). Figure 1 shows the monthly incidence series used for fitting.

**Fig. 1:**
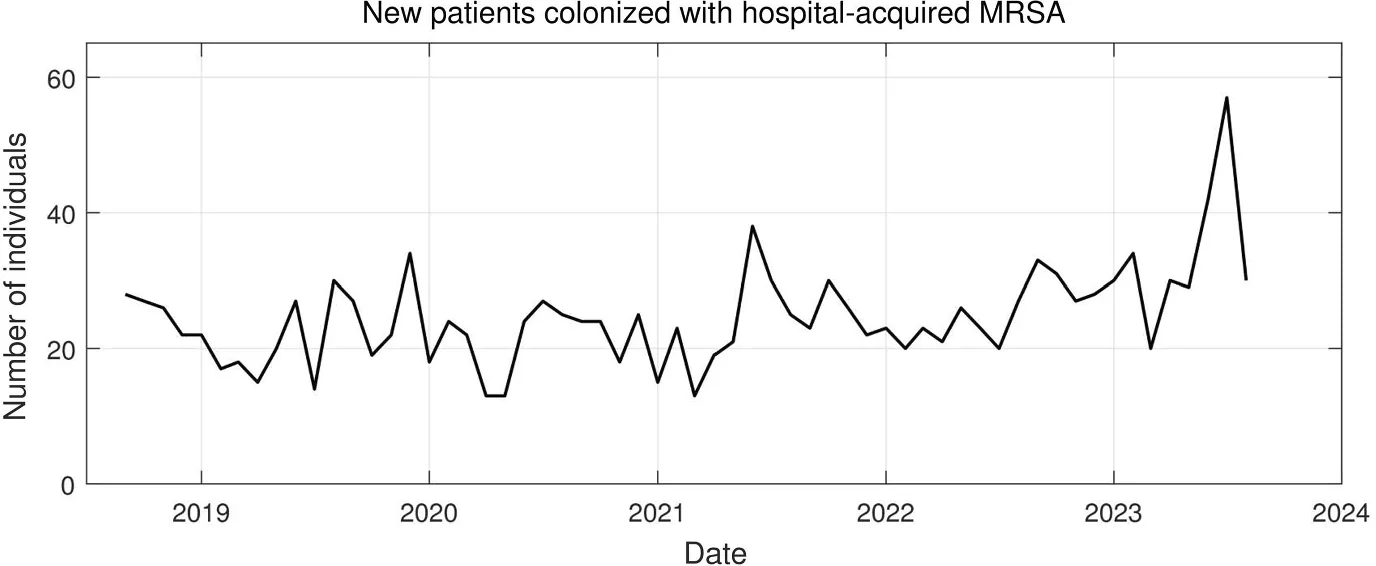
Monthly number of new patients colonized with hospital-acquired MRSA in Edmonton hospitals from September 2018 to August 2023, aggregate surveillance counts

Because monthly incidence can be noisy, we fit to cumulative incidence, defined as the running total of monthly cases. Let $D_{\textrm{monthly}}(t_k)$ denote the observed monthly number of new hospital-acquired colonizations in month *k*. The cumulative target series is

30 $$\begin{aligned} D_{\textrm{cum}}(t_k)=\sum _{j=1}^{k}D_{\textrm{monthly}}(t_j). \end{aligned}$$

For each parameter set, the model produces a corresponding cumulative prediction by tracking transitions from the susceptible state into the within-ward colonized state. Because the boundary condition is $c_W(t,0)=\lambda (t)S(t)$, the instantaneous rate of new within-ward acquisitions is λ(t)S(t). We therefore define the model-predicted cumulative number of ward acquisitions up to observation time $t_k$ as

31 $$\begin{aligned} M_{\textrm{cum}}(t_k) = \int _{0}^{t_k}\lambda (t)S(t)\,dt = \int _{0}^{t_k}c_W(t,0)\,dt. \end{aligned}$$

The corresponding model-implied incidence during month *k* is obtained by differencing successive cumulative predictions,

32 $$\begin{aligned} M_{\textrm{monthly}}(t_k) = M_{\textrm{cum}}(t_k)-M_{\textrm{cum}}(t_{k-1}). \end{aligned}$$

Thus, the cumulative model quantity compared with $D_{\textrm{cum}}(t_k)$ is explicitly the time integral of the within-ward acquisition flow λ(t)S(t).

**Sampling-based calibration and uncertainty propagation.** The final distributed-source calibration used a three-dimensional Latin Hypercube Sampling design to sample $\beta _{\max }$, $B_{50}$, and $\alpha _0$ over the parameter-search intervals reported in Table 3. The importation fraction was fixed at η=0.10 in every final calibration run and was therefore neither included in the LHS design nor estimated from the aggregate surveillance series. The final calibration evaluated $N=1{,}000{,}000$ candidate parameter sets.

For each candidate parameter set, we simulated the coupled model and calculated the cumulative number of within-ward acquisitions by integrating λ(t)S(t) over time. Model-implied monthly incidence was obtained by differencing this cumulative acquisition count at successive monthly boundaries. Behavioral acceptance was based exclusively on cumulative incidence, with parameter sets retained when cumulative $R^2\ge 0.90$. Monthly incidence was retained as a diagnostic and did not enter the behavioral acceptance criterion. A total of $N_{\textrm{acc}}=45{,}250$ parameter sets, representing 4.525% of the 1, 000, 000 evaluated sets, satisfied the cumulative acceptance criterion.

We summarize variation among the accepted parameter sets using the behavioral median and the 2.5th and 97.5th percentiles. These percentile ranges describe variation across parameter sets satisfying the behavioral acceptance criterion and are not confidence intervals, credible intervals, posterior intervals, or formal probability statements about the parameters. For each accepted parameter set, we recomputed the mechanistic $R_0$, the matched constant transmission rate, the effective dispersion parameter $k_{\textrm{disp}}$, the front-loading timing diagnostics, and the exact Stage 1 finite-threshold probability at $Z_{\textrm{crit}}=5$. Propagating all accepted sets through these calculations allows the robustness of reproductive-potential, transmission-timing, and finite-threshold conclusions to be evaluated across parameterizations consistent with the cumulative calibration target. The conditional Stage 2 ward-burden experiments are specified separately in Sect. 2.12.

### Simulation Experiments Mapped to the Two Endpoints

The analysis has two linked endpoints. The Stage 1 endpoint is the finite-threshold probability that the descendants of a single newly colonized introduction reach the operational handoff threshold $Z_{\textrm{crit}}=5$ before the transmission chain becomes extinct. Reaching this finite threshold is an operational definition used to initiate Stage 2; it is not equivalent to indefinite non-extinction and does not imply that transmission is self-sustaining, particularly when $R_0\le 1$. The Stage 2 endpoint is cumulative ward burden conditional on reaching the handoff threshold. It measures how clearance timing and immune strength affect the subsequent ward trajectory and whether a constant-transmission comparator matched to the same $R_0$ reproduces that trajectory after colonization-age timing is removed.

The two stages use different importation conventions because they answer different questions. Stage 1 evaluates the descendants of one newly colonized individual in the theoretical importation-free environment used to define $R_0$; therefore, continued external seeding is excluded from the branching calculation. Stage 2 represents the operational ICU after the handoff and retains the fixed importation fraction η=0.10 and the distributed admission source $\eta \Lambda g_A(\tau )$. Thus, Stage 1 isolates the finite transmission chain generated by one introduction, whereas Stage 2 measures conditional ward burden in the presence of continuing patient admissions and colonized importations.

At the Stage 1–Stage 2 handoff, the ward simulation is initialized with total MRSA burden $P(0)=Z_{\textrm{crit}}$, with the handoff cases placed in the ward-acquired colonized state and the remaining modeled census susceptible. To summarize the subsequent ward trajectory, we define the total colonized-or-infected census

33 $$\begin{aligned} P(t) \equiv C_A(t)+C_W(t)+I(t). \end{aligned}$$

The primary Stage 2 outcome is cumulative burden in patient-days over the simulation horizon, calculated by integrating *P*(*t*) over time. Peak burden and the compartment-specific patient-day totals are retained as supporting summaries. The Stage 2 simulations are evaluated at the componentwise behavioral-median parameter vector from the final distributed-source calibration. They are therefore representative fitted-regime scenario analyses and are not presented as behavioral confidence or credible intervals.

The constant-transmission comparator uses the same patient-flow backbone, fixed importation fraction, distributed admission source, initial handoff state, progression and removal mechanisms, and simulation horizon as the colonization-age-dependent model. Its time-invariant transmissibility $\bar{\beta }$ is chosen so that $R_0^{\textrm{const}}=R_0^{\textrm{age}}$. Consequently, the paired models differ in the timing profile of transmissibility while holding overall reproductive potential and the remaining operational assumptions fixed.

**Experiment 1: clearance timing and conditional ward burden** ($\tau _{\textrm{crit}}$). We vary the onset time of effective clearance, $\tau _{\textrm{crit}}$, which determines when immune-mediated killing changes from weak to strong in $\kappa (\tau )$. For each evaluated value of $\tau _{\textrm{crit}}$, we solve the within-host system, recompute B(τ) and E(τ), reconstruct the treated ward kernels $\beta (\tau )$ and $\alpha (\tau )$, and recompute the untreated pre-admission density $g_A(\tau )$ using $\delta _{\textrm{drug}}=0$. We then simulate the distributed-source colonization-age ward model from the common handoff state while retaining the remaining parameters at their final behavioral-median or fixed values. The primary comparison is cumulative MRSA burden in patient-days, with peak burden reported as a supporting operational summary.

**Experiment 2: immune strength and conditional ward burden** ($\kappa _{\max }$). We vary the maximum immune killing coefficient $\kappa _{\max }$ while retaining the remaining fitted coupling and ward-scale parameters at their final behavioral-median or fixed values. For each evaluated value of $\kappa _{\max }$, we recompute the within-host trajectories, reconstruct $\beta (\tau )$ and $\alpha (\tau )$, recompute the untreated pre-admission density $g_A(\tau )$ using $\delta _{\textrm{drug}}=0$, and simulate the distributed-source ward model from the common Stage 2 handoff state. Effects are summarized primarily through cumulative burden in patient-days.

**Experiment 3: timing-only contrast under matched** $R_0$. We compare the colonization-age-dependent model with its matched-$R_0$ constant-transmission comparator at the representative behavioral-median parameter vector. For each evaluated within-host scenario, we compute $R_0^{\textrm{age}}$ using (21) and select $\bar{\beta }$ using (27) so that $R_0^{\textrm{const}}=R_0^{\textrm{age}}$. Both Stage 2 ward models are then simulated from the same handoff state with the same distributed importation process. Differences in cumulative and peak burden quantify the model-implied effect of colonization-age transmission timing when overall reproductive potential and the remaining operational assumptions are held fixed. Their practical importance is assessed from their numerical magnitude rather than from statistical significance testing.

**Experiment 4: robustness to the Stage 1–Stage 2 handoff threshold.** The primary Stage 1 analysis uses $Z_{\textrm{crit}}=5$, and the exact finite-threshold probability at this value is propagated across all 45, 250 accepted behavioral parameter sets. To determine whether the representative threshold comparison depends strongly on this operational choice, we additionally recompute the exact probability at $Z_{\textrm{crit}}\in \{2,3,4,5,6,8,10,12,15,20\}$ using the behavioral-median parameter vector. This sensitivity analysis concerns finite threshold crossing before extinction and should not be interpreted as an analysis of ultimate non-extinction.

### Numerical Implementation

All computations were implemented in MATLAB. We solve the within-host system (5)–(6) on a dense colonization-age grid to obtain B(τ) and E(τ). Two within-host paths are constructed. The treated path is used to calculate the ward transmission and progression kernels $\beta (\tau )$ and $\alpha (\tau )$ through (7) and (8). The untreated pre-admission path is calculated with $\delta _{\textrm{drug}}=0$ and is used to construct the admitted-colonized age density $g_A(\tau )$ from the normalized survival function $\pi _C^{(0)}(\tau )$. Numerical checks verify that $g_A(\tau )\ge 0$, that its integral over colonization age equals one, and that the integrated distributed admission source equals the prescribed total colonized-admission rate $\eta \Lambda$.

For each candidate calibration parameter set, we simulate the distributed-source colonization-age ward model (10)–(13) using a transport discretization in colonization age coupled to time stepping for the ward variables. The boundary conditions are $c_A(t,0)=0$, $c_W(t,0)=\lambda (t)S(t)$, and i(t,0)=0, while admitted-colonized patients enter through the distributed source $\eta \Lambda g_A(\tau )$. The cumulative number of ward acquisitions is obtained by numerically integrating λ(t)S(t) over time. Cumulative $R^2$ is used for behavioral acceptance, whereas monthly incidence is calculated by differencing the cumulative trajectory at successive monthly observation times and is retained only as a diagnostic. Positivity of the ward states, normalization of $g_A(\tau )$, and conservation of the colonized-admission rate are checked numerically.

For Stage 1, we compute the mechanistic $R_0$, the matched constant transmission rate $\bar{\beta }$, and the effective dispersion parameters for the colonization-age-dependent and matched constant-transmission profiles. At $Z_{\textrm{crit}}=5$, the finite-threshold probability is calculated exactly from the negative binomial offspring transition probabilities for every accepted behavioral parameter set, with extinction and attainment of $Z_{\textrm{crit}}$ treated as absorbing outcomes. The additional sensitivity analysis over $Z_{\textrm{crit}}\in \{2,3,4,5,6,8,10,12,15,20\}$ is evaluated at the componentwise behavioral-median parameter vector. Monte Carlo branching-process realizations are used only for illustrative trajectories and numerical verification; the reported primary finite-threshold calculations are exact.

For Stage 2, we simulate the distributed-source ward model conditional on the common handoff state $P(0)=Z_{\textrm{crit}}$ using the componentwise behavioral-median parameter vector from the final calibration. For each clearance-timing or immune-strength scenario, we recompute the within-host trajectories, reconstruct $\beta (\tau )$ and $\alpha (\tau )$, and recompute $g_A(\tau )$ from the corresponding untreated pre-admission path with $\delta _{\textrm{drug}}=0$. The matched constant-transmission comparator uses the same handoff state, patient-flow parameters, distributed admission source, progression and removal mechanisms, and simulation horizon, with $\bar{\beta }$ selected through (27) so that $R_0^{\textrm{const}}=R_0^{\textrm{age}}$. Cumulative burden is calculated by numerically integrating *P*(*t*) over the simulation horizon, and peak burden is obtained as the maximum of *P*(*t*) over that horizon.

Where distributions of derived quantities are reported, the median and the 2.5th and 97.5th percentiles are calculated across the accepted behavioral parameter sets. These summaries describe variation among parameterizations satisfying the cumulative calibration criterion and are not confidence intervals, credible intervals, or posterior intervals. We now turn to the Results, which quantify the endpoints under the four experiments described in Sect. 2.12.

## Results and Discussion

The research question has two linked parts. The first asks how colonization-age-dependent, front-loaded transmissibility and the resulting mechanistic heterogeneity affect the probability that a single introduction reaches the operational handoff threshold $Z_{\textrm{crit}}$ before extinction. The second asks, conditional on reaching that threshold, how clearance timing and immune strength affect cumulative ward burden and whether a constant-transmission model matched to the same $R_0$ reproduces those burden outcomes after colonization-age timing is removed. We present the results in this order.

We first evaluate calibration of the distributed-source model to the Edmonton hospital-acquired MRSA series. We then quantify reproductive potential, the colonization-age timing of that potential, and the robustness of these quantities across the accepted behavioral parameter sets. Next, we evaluate the finite-threshold probability used for the Stage 1 handoff. Finally, we examine conditional ward burden in Stage 2, including the matched-$R_0$ comparison and the effects of clearance timing and immune strength.

### Model Calibration and Behavioral Parameter Sets

Using the Edmonton calibration target described in Sect. 2.11, we evaluated $N=1{,}000{,}000$ Latin Hypercube Sampling parameter sets for the final distributed-source model. The final refit did not estimate four parameters simultaneously: the importation fraction was fixed at η=0.10, and only $\beta _{\max }$, $B_{50}$, and $\alpha _0$ were sampled. A total of $N_{\textrm{acc}}=45{,}250$ parameter sets, representing 4.525% of all evaluated sets, satisfied the behavioral acceptance criterion of cumulative $R^2\ge 0.90$. Thus, the absolute number of accepted sets reflects the large number of candidate sets evaluated, whereas the accepted proportion was 4.525%. Because the cumulative target smooths month-to-month variation, the accepted sets are interpreted as behaviorally admissible parameter combinations rather than as evidence that each sampled parameter is uniquely identified. The maximum cumulative coefficient of determination was $R^2=0.995865$, reported as 0.9959 in Fig. 2.

**Fig. 2:**
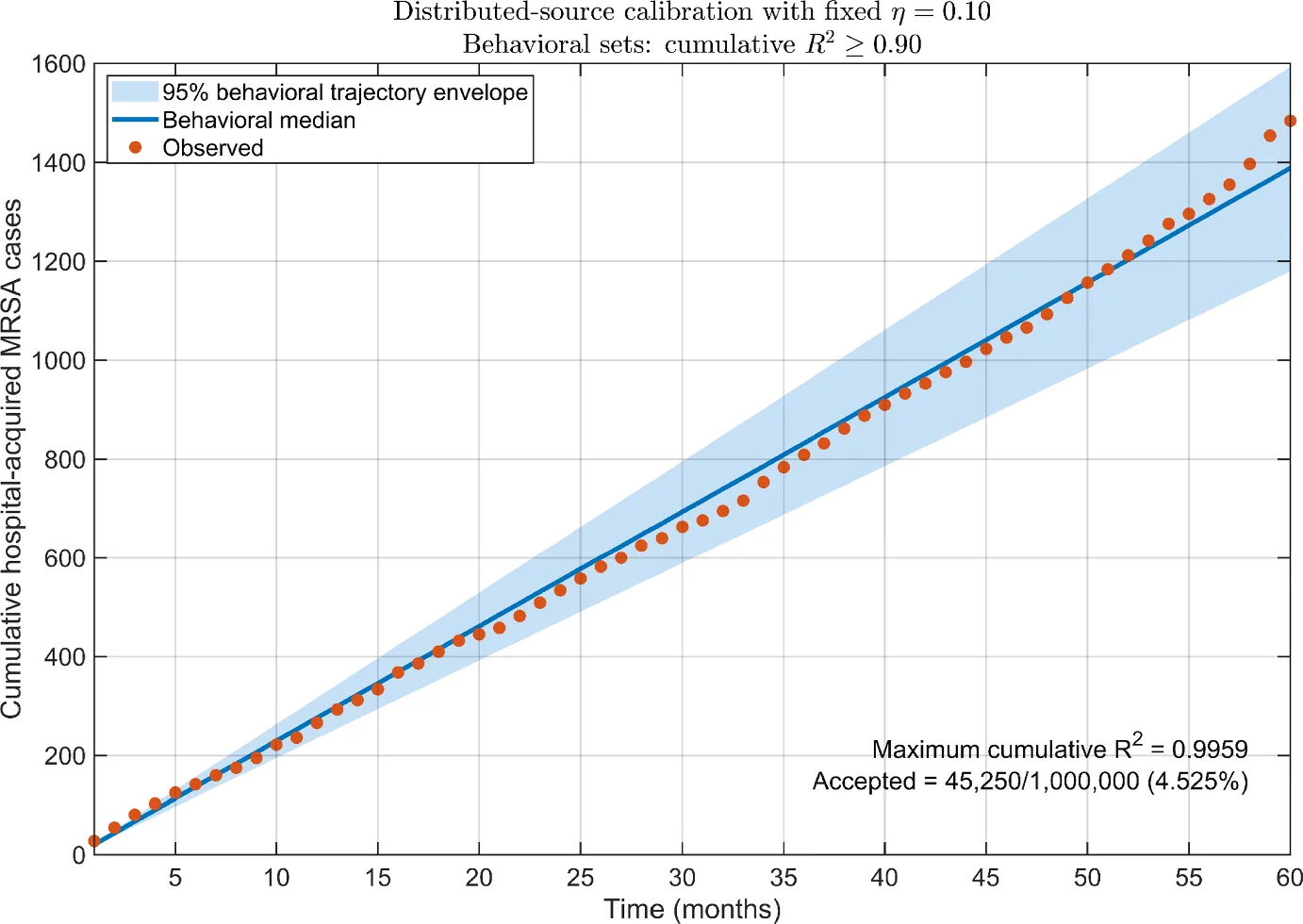
Cumulative calibration of the final distributed-source model to the Edmonton hospital-acquired MRSA colonization series. Orange points show the observed cumulative counts $D_{\textrm{cum}}(t_k)$, the solid blue curve shows the behavioral median, and the shaded region shows the pointwise 95% behavioral trajectory envelope, defined by the 2.5th and 97.5th percentiles across the 45, 250 accepted parameter sets. Behavioral acceptance required cumulative $R^2\ge 0.90$, and the importation fraction was fixed at η=0.10. The annotation reports the maximum cumulative $R^2$ and the accepted fraction. The shaded region is a behavioral trajectory range and not a confidence or credible interval (Color figure online)

Figure 2 compares the observed cumulative hospital-acquired MRSA counts with the behavioral median and the pointwise 95% behavioral trajectory envelope across accepted parameter sets. The envelope is defined by the 2.5th and 97.5th percentiles of the accepted trajectories at each observation time. It is therefore a behavioral-set range and not a confidence interval, credible interval, or posterior interval.

The sampled parameters in Table 4 determine the colonization-age mechanisms used in all downstream calculations. The parameters $\beta _{\max }$ and $B_{50}$ determine the upper scale and bacterial-load dependence of transmissibility through $\beta (\tau )$, whereas $\alpha _0$ determines how bacterial burden contributes to the progression hazard $\alpha (\tau )$. The fixed value η=0.10 determines the total inflow of colonized admissions, while the normalized density $g_A(\tau )$ distributes that inflow across pre-admission colonization ages.

**Table 4:** Final calibration values for the distributed-source model.

Parameter	Symbol	Behavioral median or fixed value	95% behavioral range
Maximum transmissibility	$\beta _{\max }$	$0.2263~\textrm{day}^{-1}$	$[0.1710,\,0.2601]~\textrm{day}^{-1}$
Half-saturation bacterial load	$B_{50}$	$5.22\times 10^{7}~\mathrm {CFU\,ml}^{-1}$	$[3.11\times 10^{6},\,9.76\times 10^{7}]~\mathrm {CFU\,ml}^{-1}$
Progression scaling from colonization to infection	$\alpha _0$	$4.98\times 10^{-8}~\mathrm {ml\,CFU}^{-1}\textrm{day}^{-1}$	$[2.47\times 10^{-9},\,9.75\times 10^{-8}]~\mathrm {ml\,CFU}^{-1}\textrm{day}^{-1}$
Importation fraction among admissions	η	0.10 (fixed)	Not applicable

For each sampled coupling parameter, the table reports the behavioral median and the 95% behavioral range, defined by the 2.5th and 97.5th percentiles across the $N_{\textrm{acc}}=45{,}250$ parameter sets satisfying cumulative $R^2\ge 0.90$. The importation fraction η=0.10 was fixed for the final refit and was not sampled

The behavioral median $\beta _{\max }=0.2263~\textrm{day}^{-1}$ is the upper bound approached by the transmission kernel when $B(\tau )\gg B_{50}$; it is not an unconditional acquisition rate. It enters the frequency-dependent force of colonization together with the colonization-age composition of the ward.

34 $$\begin{aligned} \lambda (t)=\frac{1}{N(t)}\int _0^\infty \beta \!\left( B(\tau )\right) \,[c_A(t,\tau )+c_W(t,\tau )+i(t,\tau )]\,d\tau . \end{aligned}$$

Consequently, realized acquisitions also depend on the susceptible population through the incidence term λ(t)S(t). The behavioral median $B_{50}=5.22\times 10^7~\mathrm {CFU\,ml}^{-1}$ is the burden at which transmissibility reaches one half of $\beta _{\max }$. With the parsimonious Hill coefficient h=1 used here, lower bacterial burdens make nonzero but down-weighted contributions relative to higher-burden phases. The behavioral median $\alpha _0=4.98\times 10^{-8}~\mathrm {ml\,CFU}^{-1}\textrm{day}^{-1}$ links bacterial burden to the colonization-age-dependent progression hazard.

The sensitivity refits revealed an important identifiability limitation. Refits with free η and with η fixed at 0.01, 0.05, and 0.10 produced maximum cumulative $R^2$ values between 0.995758 and 0.996314. The near-equivalence of these fits shows that the aggregate cumulative series cannot uniquely distinguish colonized admissions from within-ward acquisitions. For epidemiological context, a nationally representative U.S. survey estimated nasal MRSA colonization at 1.5% in 2003–2004, whereas an urban-hospital admission study found MRSA nasal colonization in 7.3% of admitted patients (Gorwitz et al. 2008; Hidron et al. 2005). These estimates illustrate that carriage prevalence depends strongly on the population and setting, but neither estimate directly determines η for the modeled ICU because admission prevalence also depends on referral patterns, surveillance period, anatomical sites sampled, and microbiological detection methods. We fixed η=0.10 to retain a non-negligible but bounded importation pathway while removing η as an additional fitting degree of freedom. Accordingly, η=0.10 is a transparent modeling assumption and not an estimated admission-screening prevalence. Independent admission-screening data from the modeled institutions would be required to estimate this quantity directly.

Monthly incidence was retained as a diagnostic and was not used for behavioral acceptance. At the parameter set with the maximum cumulative $R^2$, the monthly root-mean-square error was 7.5309 cases per month and the monthly $R^2$ was -0.0675. The final model therefore reproduces the long-term cumulative trend closely but does not reproduce all month-to-month fluctuations. This limitation prevents the high cumulative $R^2$ from being interpreted as equally strong monthly predictive performance.

### Reproductive Potential and Front-Loaded Colonization-Age Timing

We next quantify the reproductive potential implied by the final distributed-source calibration and identify the colonization-age window that contributes most of that potential. In this framework, an introduced colonized patient is not uniformly infectious. Transmissibility varies with colonization age τ through the within-host bacterial burden B(τ) and is weighted by the probability that the patient remains present in a transmissible colonized or infected state at that colonization age.

For the representative accepted-median parameter combination, the age-dependent reproduction number is calculated from

35 $$\begin{aligned} \begin{aligned} R_0^{\textrm{age}}&= \int _0^\infty \beta (\tau ) \left[ \pi _C(\tau )+\pi _I(\tau )\right] \,d\tau \\&= \int _0^\infty \beta _{\max } \frac{B(\tau )}{B(\tau )+B_{50}} \left[ \pi _C(\tau )+\pi _I(\tau )\right] \,d\tau . \end{aligned} \end{aligned}$$

Numerical evaluation using the final accepted-median coupling parameters gives $R_0^{\textrm{age}}=0.907957$. The terminal transmissible-state probability at the end of the numerical integration was $7.105\times 10^{-14}$, confirming that the remaining contribution from the numerical tail was negligible. The matched constant-transmission comparator has the same reproduction number, $R_0^{\textrm{const}}=0.907957$, with matched constant transmission rate $\bar{\beta }=0.160736~\textrm{day}^{-1}$.

**Fig. 3:**
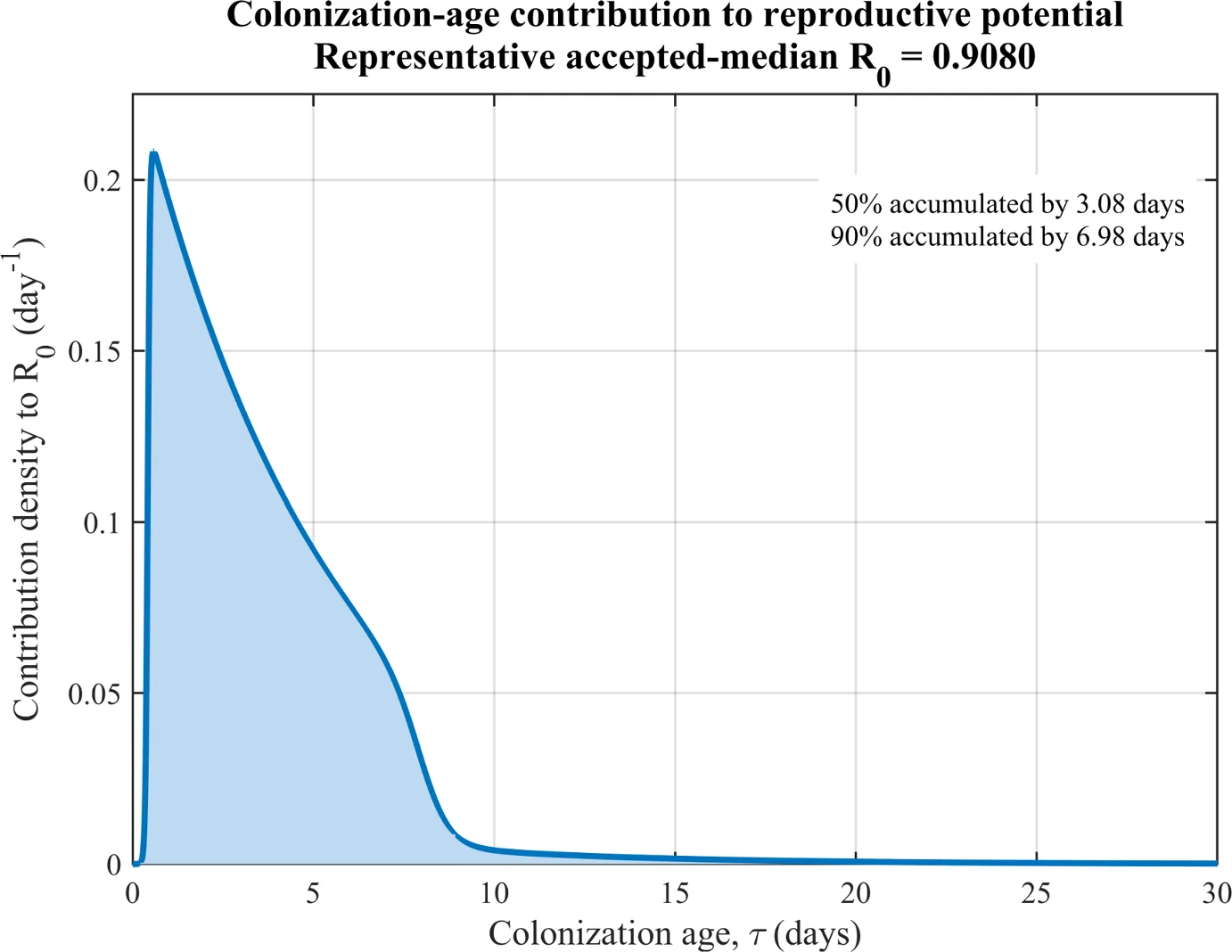
Colonization-age contribution to the reproduction number for the final representative accepted-median parameter combination. The curve shows $R_{\textrm{new}}(\tau )=\beta (\tau )[\pi _C(\tau )+\pi _I(\tau )]$, and the shaded area under the complete curve equals $R_0^{\textrm{age}}=0.907957$. A total of 50% of the reproductive potential is accumulated by 3.0814 days and 90% by 6.9833 days. The representative calculation is subcritical because $R_0<1$ (Color figure online)

Because the representative value is below one, this accepted-median calculation is subcritical. Consequently, the ultimate non-extinction probability is zero for both the age-dependent and matched constant profiles. The operational Stage 1 probability of reaching a finite handoff threshold is nevertheless positive and is reported separately in Sect. 3.4. The importation fraction η=0.10 is not included in this $R_0$ calculation because $R_0$ is evaluated at the theoretical importation-free disease-free state. Imported colonized admissions provide repeated external seeding in the fitted ward simulations but are not counted as secondary cases generated by an introduced individual.

Fig. 3 shows the colonization-age-specific contribution $R_{\textrm{new}}(\tau )=\beta (\tau )[\pi _C(\tau )+\pi _I(\tau )]$. The area under the complete contribution curve equals $R_0^{\textrm{age}}=0.907957$. Although the transmission rate is initially negligible, it rises rapidly as bacterial burden increases and then declines sharply around the effective-clearance transition. This produces a concentrated early transmission window rather than a constant accumulation of reproductive potential throughout the transmissible stay.

The timing calculation confirms this front-loading. Under the age-dependent profile, 50% of $R_0$ is accumulated by 3.0814 days and 90% by 6.9833 days. Under the matched constant profile, the corresponding times are 3.8150 and 13.0483 days. Thus, matching total $R_0$ does not match the timing at which reproductive potential is generated.

Propagation across all 45, 250 accepted behavioral parameter sets shows that the behavioral median of $R_0^{\textrm{age}}$ is 0.917464, with a 95% behavioral range of [0.814593,1.032975]. A total of 41, 838 accepted sets, representing 92.460%, have $R_0<1$, whereas 3, 412 sets, representing 7.540%, have $R_0>1$. Subcriticality therefore strongly predominates across the accepted ensemble, although it is not universal. These percentiles describe the accepted behavioral sets and are not confidence or credible intervals.

### Mechanistically Induced Heterogeneity and an Effective Dispersion Parameter

The Stage 1 ignition endpoint depends on both the mean number of secondary colonizations, measured by $R_0$, and how unevenly those secondary colonizations are distributed across patients. Even when two transmission profiles have the same $R_0$, an early chain can die out if the first few introduced patients happen to generate few or no secondary colonizations. We therefore carry the patient-level variability generated by the mechanistic model into the Stage 1 branching process.

Let *T* be the random first-exit time at which an introduced patient leaves the union of the transmissible colonized and infected states. From the survival equations described under the paragraph heading *Single-individual survival in transmissible states* in Section 2.6, the probability that the patient remains transmissible at colonization age τ is

$$ S_T(\tau )=\Pr (T>\tau )=\pi _C(\tau )+\pi _I(\tau ), $$

and hence

$$ F_T(\tau )=\Pr (T\le \tau )=1-S_T(\tau ). $$

The random cumulative transmission potential is

$$ V=\int _0^T\beta (\tau )\,d\tau . $$

Thus, individuals who leave the transmissible states early tend to have small values of *V*, whereas individuals who remain through more of the transmissible window tend to have larger values.

To generate the virtual-individual sample, we first calculated the deterministic functions $\beta (\tau )$ and $F_T(\tau )$ on the common colonization-age grid. For each of $N_{\textrm{VP}}=10^6$ virtual individuals, we independently drew $U_i\sim \textrm{Uniform}(0,1)$, obtained

$$ T_i=F_T^{-1}(U_i) = \inf \left\{ \tau :F_T(\tau )\ge U_i\right\} , $$

and calculated

$$ v_i=\int _0^{T_i}\beta (\tau )\,d\tau . $$

The resulting values $\{v_i\}_{i=1}^{N_{\textrm{VP}}}$ form a Monte Carlo sample from the distribution of *V*. Each virtual trajectory therefore uses the exit-time distribution generated by the same discharge, decolonization, progression, recovery, and mortality mechanisms represented in $\pi _C(\tau )$ and $\pi _I(\tau )$.

The transmission potential *V* is continuous, whereas the actual offspring count *Y* is a nonnegative integer. Conditional on a particular realized value V=v, we assume

$$ Y\mid V=v\sim \operatorname {Poisson}(v). $$

In other words, once an individual-specific value *v* has been obtained, that individual’s offspring count is Poisson distributed with mean *v*. Because *V* varies across individuals, the unconditional distribution of *Y* is a mixed-Poisson distribution rather than a single Poisson distribution with one fixed mean. Consequently,

$$ \textrm{E}[Y]=\textrm{E}[V]=R_0 $$

and

$$ \textrm{Var}(Y) = \textrm{E}[V]+\textrm{Var}(V) = R_0+\textrm{Var}(V). $$

We represent this mixed-Poisson offspring distribution by an effective negative binomial distribution satisfying

$$ \textrm{E}[Y]=R_0, \qquad \textrm{Var}(Y)=R_0+\frac{R_0^2}{k_{\textrm{disp}}}. $$

Matching the two variances gives

$$ k_{\textrm{disp}} = \frac{\bigl (\textrm{E}[V]\bigr )^2}{\textrm{Var}(V)} = \frac{R_0^2}{\textrm{Var}(V)}. $$

Greater between-individual variation in cumulative transmission potential therefore produces a smaller effective dispersion parameter and greater offspring heterogeneity.

For the representative parameter-median combination in the final distributed-source analysis, the age-dependent profile gives $R_0=0.907957$ and $k_{\textrm{disp}}^{\textrm{age}}=1.855332$. The matched constant-transmission profile has the same reproduction number, $R_0=0.907957$, but gives $k_{\textrm{disp}}^{\textrm{const}}=1.010124$. Thus, matching the mean reproductive potential does not reproduce the variance generated by the colonization-age-dependent transmission profile. The virtual-individual samples provide diagnostic representations of the distributions of individual transmission potential, whereas the reported values of $R_0$ and $k_{\textrm{disp}}$ are obtained from the corresponding mechanistic moment calculations.

Figure 4 shows that the two transmission profiles have the same mean individual transmission potential but different distributional shapes. Both distributions place appreciable probability near V=0 because some introduced patients leave the transmissible states before accumulating substantial transmission potential. Under the colonization-age-dependent profile, the additional concentration near V≈1.8 arises because $\beta (\tau )$ declines sharply around the effective-clearance transition. Consequently, a range of longer transmissible-state durations produces relatively little additional transmission potential and is compressed into a narrow range of *V*. By contrast, under the matched constant profile, transmission potential accumulates as $V=\bar{\beta }T$, producing a more extended right tail. Accordingly, the matched constant profile has the larger variance, $\textrm{Var}(V)=0.816124$ compared with 0.444334 for the age-dependent profile, and therefore the smaller effective dispersion parameter. Thus, although the two profiles have identical $R_0$, they do not generate the same degree or pattern of individual-level heterogeneity.

**Fig. 4:**
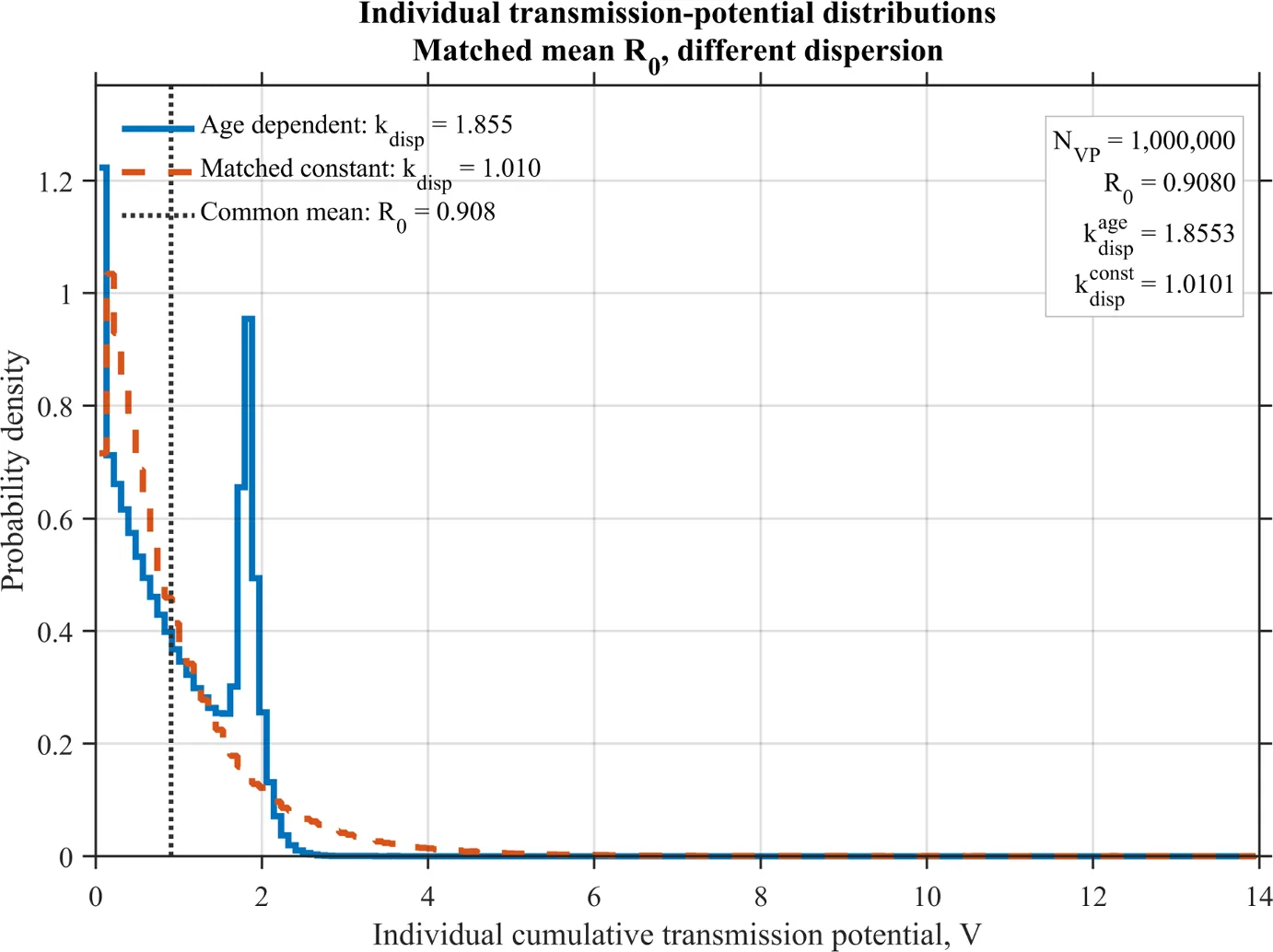
Probability-density distributions of individual cumulative transmission potential *V* for $N_{\textrm{VP}}=10^6$ virtual individuals under the final accepted-median colonization-age-dependent transmission profile and its matched-$R_0$ constant-transmission comparator. The solid blue curve represents the age-dependent profile, the dashed orange curve represents the matched constant profile, and the vertical dotted line marks their common mean, $R_0=0.907957$. Mechanistic moment matching gives $k_{\textrm{disp}}^{\textrm{age}}=1.855332$ and $k_{\textrm{disp}}^{\textrm{const}}=1.010124$. The concentration in the age-dependent distribution near V≈1.8 results from the sharp reduction in transmissibility around the effective-clearance transition, whereas the constant profile has a longer right tail. The virtual-individual distributions are Monte Carlo diagnostic representations; the reported moments and dispersion parameters are obtained from the mechanistic moment calculations (Color figure online)

### Ignition Risk: Finite-Threshold Crossing and Robustness to the Handoff Threshold

We now answer the first endpoint in the research question: starting from one introduced colonized patient, what is the probability that its descendants reach the operational handoff threshold before the transmission chain becomes extinct? As specified for Stage 1 in Sect. 2.8, we use a Galton–Watson branching process with negative binomial offspring:

$$ Z_0=1,\qquad Z_{n+1}=\sum _{i=1}^{Z_n}Y_{n,i}, \qquad Y\sim \textrm{NB}\!\left( k_{\textrm{disp}},R_0\right) . $$

The negative binomial distribution is parameterized to have mean $R_0$ and variance $R_0+R_0^2/k_{\textrm{disp}}$.

For the representative parameter-median combination in the final distributed-source analysis, the colonization-age-dependent profile gives $R_0=0.907957$ and $k_{\textrm{disp}}^{\textrm{age}}=1.855332$. Its matched constant-transmission comparator has the same reproduction number, $R_0=0.907957$, but has $k_{\textrm{disp}}^{\textrm{const}}=1.010124$. Because the common reproduction number is below one, both branching processes are subcritical. Their ultimate extinction probabilities are therefore one, and their ultimate non-extinction probabilities are zero.

We classify a chain as having reached the operational Stage 1 handoff when it attains $Z_{\textrm{crit}}=5$ before extinction. This is a finite-threshold event and must not be interpreted as indefinite establishment. The exact absorbing-state calculation gives

$$ P_{\textrm{thr}}^{\textrm{age}}(5)=0.114659 $$

for the colonization-age-dependent profile and

$$ P_{\textrm{thr}}^{\textrm{const}}(5)=0.117074 $$

for the matched constant profile. Thus, approximately 11.466% and 11.707% of introductions, respectively, reach five before extinction. The age-dependent minus constant difference is -0.002415, or approximately -0.241 percentage points. Correspondingly, approximately 88.534% of introductions under the age-dependent profile become extinct before reaching five. Figure 5 shows illustrative examples of trajectories that reach the handoff threshold and trajectories that become extinct before reaching it.

**Fig. 5:**
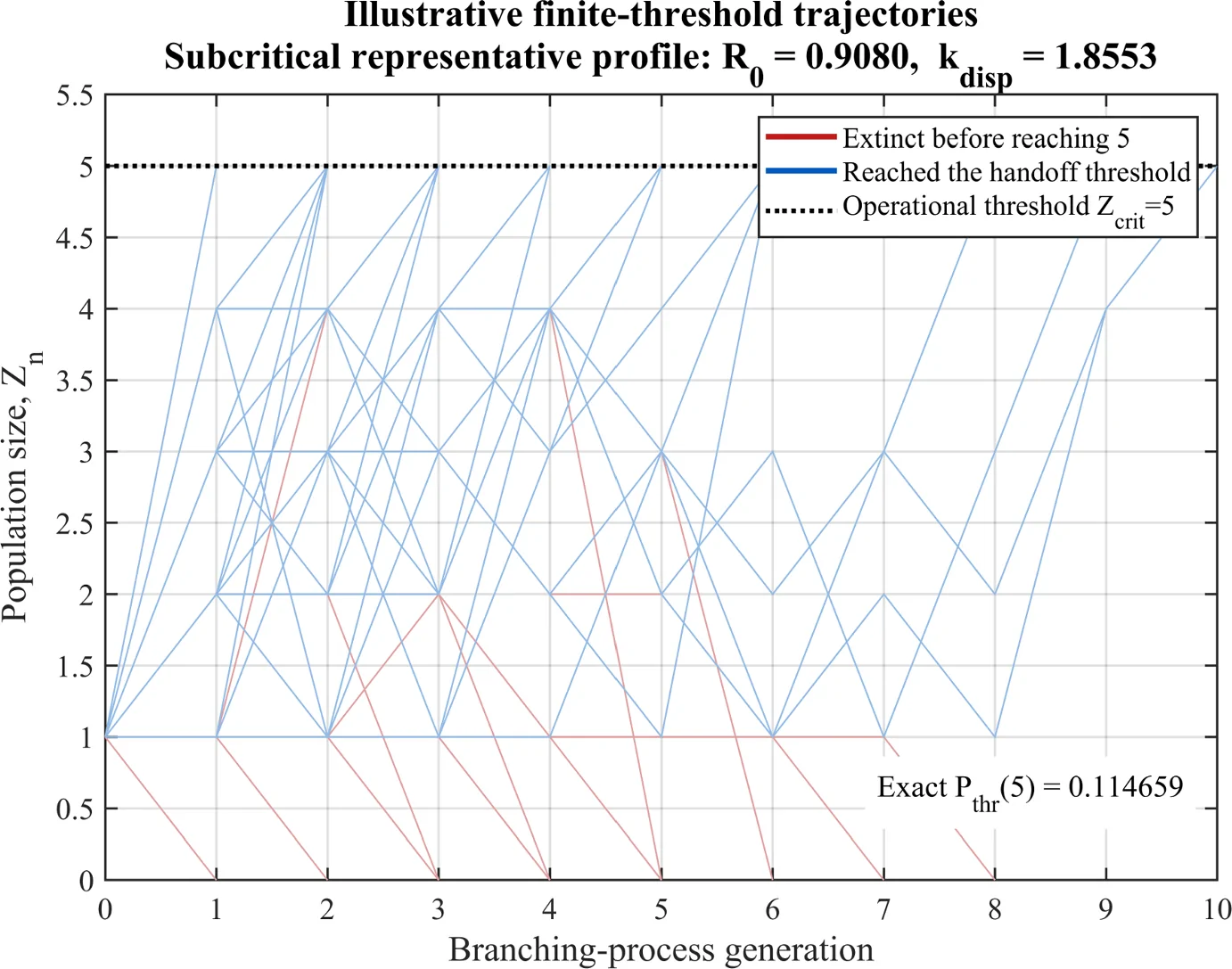
Illustrative Stage 1 branching-process trajectories for the final colonization-age-dependent transmission profile, starting from $Z_0=1$ and using the operational handoff threshold $Z_{\textrm{crit}}=5$. Red trajectories become extinct before reaching five, whereas blue trajectories reach five and are classified as finite-threshold crossings. Equal numbers of the two outcomes are displayed only for visual clarity and are not used to calculate the probability. The representative calculation uses $R_0=0.907957$ and $k_{\textrm{disp}}^{\textrm{age}}=1.855332$. The exact probability of reaching five before extinction is $P_{\textrm{thr}}^{\textrm{age}}(5)=0.114659$. Because $R_0<1$, reaching the finite threshold does not imply indefinite non-extinction (Color figure online)

Because $Z_{\textrm{crit}}$ is an operational choice, we repeated the exact calculation over

$$ Z_{\textrm{crit}}\in \{2,3,4,5,6,8,10,12,15,20\}. $$

For the representative parameter-median combination, the age-dependent threshold probability decreases from 0.326327 at $Z_{\textrm{crit}}=2$ to 0.008195 at $Z_{\textrm{crit}}=20$. The corresponding matched-constant probability decreases from 0.302195 to 0.011849. Both probabilities therefore approach zero as the threshold increases, as required for subcritical branching processes.

The relative ordering of the two profiles changes with the operational threshold. The age-dependent probability is higher at $Z_{\textrm{crit}}=2$, 3, and 4, but the difference reverses between thresholds four and five. At $Z_{\textrm{crit}}\ge 5$ over the examined grid, the matched constant profile has a slightly higher threshold-crossing probability. This comparison shows that matching $R_0$ does not reproduce the complete finite-threshold behavior, but it also shows that the direction of the difference is not independent of the selected handoff threshold.

This threshold dependence follows from the distinct offspring distributions shown in Fig. 4. Under constant transmission, cumulative transmission potential continues to increase throughout a patient’s transmissible stay, producing a longer right tail, a larger variance, and a smaller effective dispersion parameter. The age-dependent profile concentrates more transmission opportunity earlier and has a shorter right tail. The probability of crossing a particular finite threshold depends on the complete offspring distribution rather than on $R_0$ or $k_{\textrm{disp}}$ alone; consequently, a smaller dispersion parameter does not imply the same ordering at every finite threshold.

Propagation across all 45, 250 accepted behavioral parameter sets further qualifies the representative calculation. The behavioral median of the age-dependent reproduction number is 0.917464, with a 95% behavioral range of [0.814593,1.032975]. A total of 41, 838 accepted sets, representing 92.460%, have $R_0<1$, whereas 3, 412 sets, representing 7.540%, have $R_0>1$. Thus, subcriticality is strongly predominant across the accepted ensemble but is not universal.

At $Z_{\textrm{crit}}=5$, the age-dependent threshold probability has behavioral median 0.119479 and 95% behavioral range [0.076856,0.180713]. The matched constant probability has behavioral median 0.120843 and range [0.082411,0.166780]. The age-dependent minus constant difference has median -0.001060 and range [-0.007436,0.013802], which crosses zero. The age-dependent probability is higher in 18, 861 accepted sets (41.682%) and lower in 26, 389 sets (58.318%). Therefore, the sign of the finite-threshold difference at five is not robust across accepted fits, although its representative and ensemble-median magnitudes are small. These percentile ranges are behavioral-set ranges and are not confidence or credible intervals (Fig. 6).

**Fig. 6:**
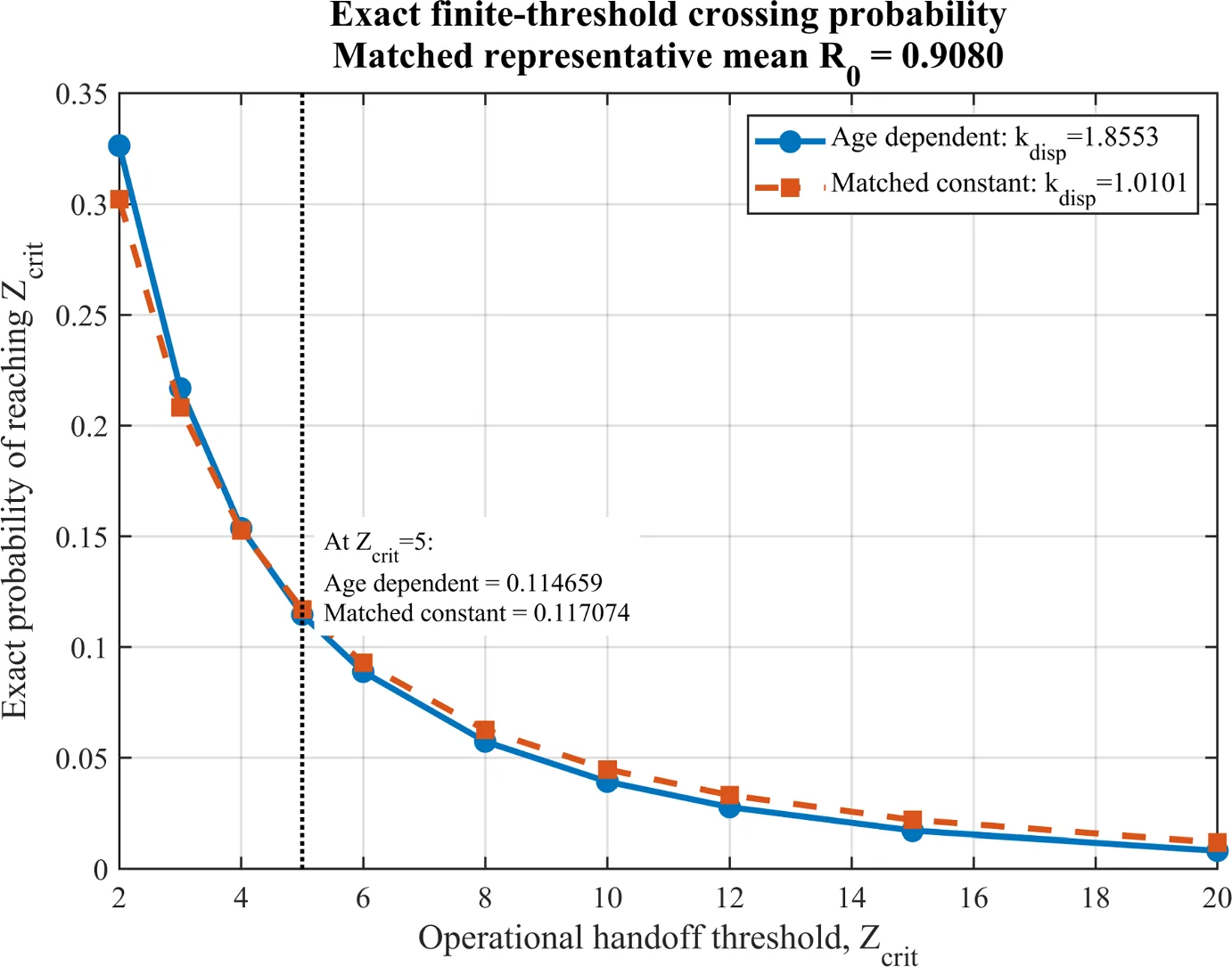
Exact probability of reaching the operational Stage 1 handoff threshold $Z_{\textrm{crit}}$ before extinction for the representative colonization-age-dependent transmission profile and its matched-$R_0$ constant-transmission comparator. Both profiles have $R_0=0.907957$, while their effective dispersion parameters are $k_{\textrm{disp}}^{\textrm{age}}=1.855332$ and $k_{\textrm{disp}}^{\textrm{const}}=1.010124$. At $Z_{\textrm{crit}}=5$, the exact probabilities are 0.114659 and 0.117074, respectively. The ordering reverses between thresholds four and five, and both curves approach zero as the threshold increases because the representative branching processes are subcritical (Color figure online)

### Conditional Ward Burden Under Delayed Clearance: Effect of $\tau _{\textrm{crit}}$ Conditional on Establishment

We next address the second endpoint of the research question: ward burden conditional on reaching the operational Stage 1 handoff threshold. The Stage 2 simulations begin from $P(0)=Z_{\textrm{crit}}=5$ colonized-or-infected patients and retain the final distributed admission source $\eta \Lambda g_A(\tau )$ with η=0.10. We report two complementary measures of total MRSA burden. The maximum concurrent total burden is $\max _{0\le t\le T_{\textrm{sim}}}P(t)$, where $P(t)=C_A(t)+C_W(t)+I(t)$. The cumulative total burden is $\int _0^{T_{\textrm{sim}}}P(t)\,dt$, expressed in patient-days. Thus, panel (b) of Fig. 7 reports total colonized-or-infected patient-days and not infection-only patient-days.

We varied the onset of effective clearance, $\tau _{\textrm{crit}}$, from 5 to 15 days. For each delay, the corresponding within-host path and colonization-age-dependent kernels were recalculated. The matched constant transmission rate $\bar{\beta }$ was then selected separately at that delay so that the age-dependent and constant profiles had exactly the same $R_0$. Consequently, $R_0$ is matched within every paired comparison but is not held fixed across the clearance-delay sweep. Delaying clearance increased the paired value of $R_0$ from 0.627881 at $\tau _{\textrm{crit}}=5$ days to 1.078492 at $\tau _{\textrm{crit}}=15$ days.

**Fig. 7:**
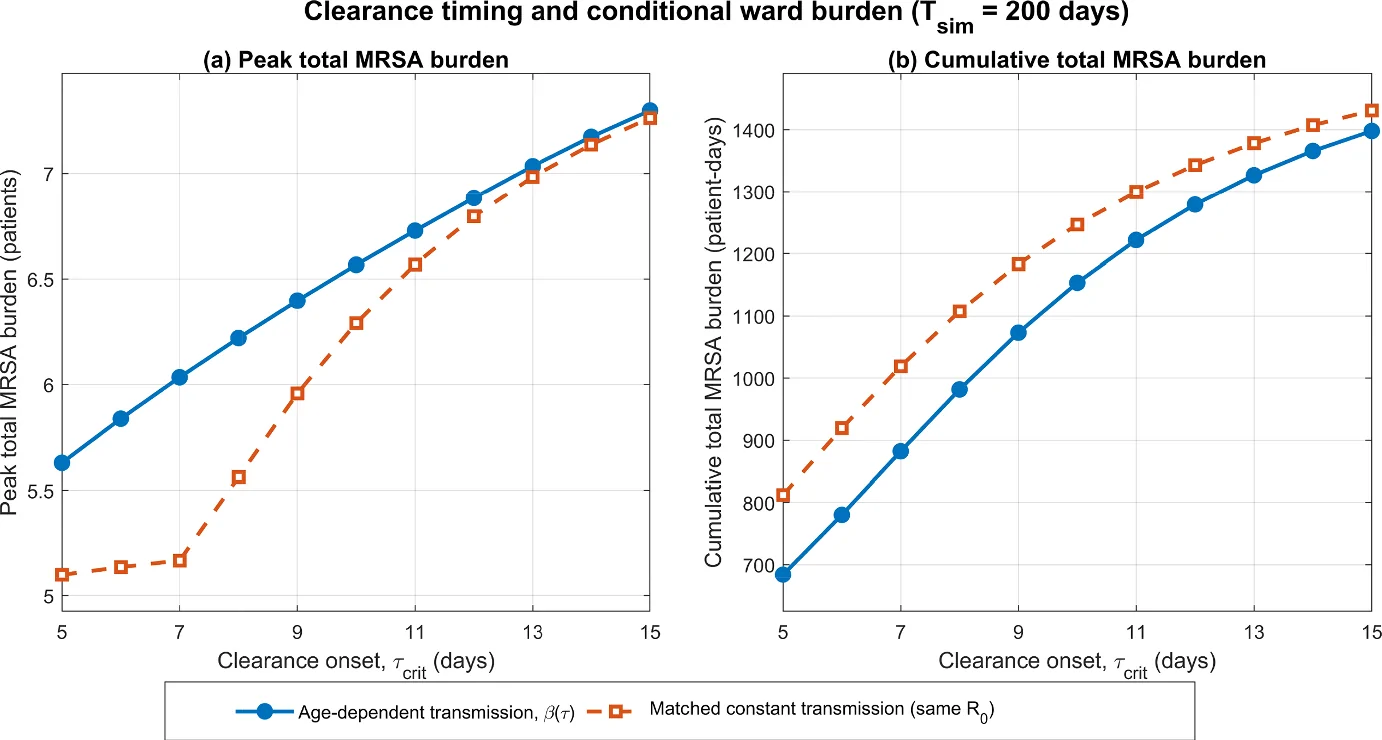
Conditional ward burden as clearance onset varies in the final distributed-source analysis. Stage 2 is initialized at the operational handoff threshold $P(0)=Z_{\textrm{crit}}=5$ and retains the distributed colonized-admission source with η=0.10. At every value of $\tau _{\textrm{crit}}$, the matched constant rate $\bar{\beta }$ is selected to give exactly the same $R_0$ as the age-dependent profile $\beta (\tau )$. The matching is performed separately at each delay; therefore, $R_0$ is matched within each pair but varies across the delay scenarios. Panel (a) shows the maximum concurrent total MRSA burden, $\max _{0\le t\le T_{\textrm{sim}}}P(t)$. Panel (b) shows the cumulative total MRSA burden, $\int _0^{T_{\textrm{sim}}}P(t)\,dt$, in patient-days, where $P(t)=C_A(t)+C_W(t)+I(t)$ and $T_{\textrm{sim}}=200$ days. The bend in the matched constant curve in panel (a) reflects a change from an early post-handoff maximum to a later ward-level maximum. Each plotted point is the result of a separate paired ward simulation (Color figure online)

In Fig. 7(a), peak total MRSA burden increases with delayed clearance under both transmission profiles. For the age-dependent profile, the peak increases from 5.629 patients at $\tau _{\textrm{crit}}=5$ days to 6.398 patients at the baseline value of 9 days and 7.298 patients at 15 days. The corresponding matched constant-profile peaks are 5.100, 5.958, and 7.264 patients. The age-dependent peak is therefore higher at every examined delay. At the baseline delay, the profiles have the same $R_0=0.907957$, but the age-dependent peak exceeds the matched constant peak by 0.439 patient, or 7.372%. The largest separation occurs at $\tau _{\textrm{crit}}=7$ days, where the difference is 0.866 patient, or 16.764%, whereas the difference decreases to 0.035 patient, or 0.478%, at 15 days.

The bend in the matched constant peak-burden curve between $\tau _{\textrm{crit}}=7$ and 8 days is a genuine consequence of taking the maximum over ward calendar time. At the shorter delays, the maximum is attained during the early post-handoff transient, whereas at the longer delays it is attained during a later ward-level peak. The identity of the local maximum defining $\max _tP(t)$ therefore changes between these delay scenarios. The bend does not represent a discontinuity in an individual ward trajectory or a plotting artifact.

Fig. 7(b) shows a different ordering for cumulative total burden. The age-dependent profile produces fewer total MRSA patient-days than the matched constant profile at every examined delay. At $\tau _{\textrm{crit}}=5$ days, the cumulative burdens are 683.8 and 812.0 patient-days for the age-dependent and matched constant profiles, respectively, corresponding to an age-dependent-minus-constant difference of -128.2 patient-days, or -15.790%. At the baseline delay of 9 days, the corresponding values are 1073.2 and 1183.3 patient-days, giving a difference of -110.1 patient-days, or -9.304%. At 15 days, the values are 1398.0 and 1430.9 patient-days, giving a difference of -32.9 patient-days, or -2.301%. The cumulative-burden curves therefore converge as clearance is delayed but do not cross over the examined range.

These paired results show that matching $R_0$ does not reproduce either the temporal concentration or the accumulated magnitude of conditional ward burden. The fitted age-dependent profile concentrates transmission opportunity earlier, producing a higher short-term peak but fewer total MRSA patient-days over the 200-day horizon. The matched constant profile produces a lower peak but distributes transmission opportunity more persistently, resulting in greater cumulative total burden. Because Stage 2 retains the distributed source of colonized admissions, positive burden in pairs with $R_0<1$ should not be interpreted as evidence of a positive ultimate non-extinction probability for a single introduction. Here, $T_{\textrm{sim}}=200$ days denotes the ward calendar-time simulation horizon and is distinct from the colonization-age integration cutoff $T_{\textrm{end}}$ used to calculate $R_0$.

### Immune Strength and Conditional Ward Burden with Fixed Clearance Timing

We next examine how immune strength affects reproductive potential and conditional ward burden when the timing of effective clearance is held fixed. These calculations use the final representative accepted-median parameter combination. We fix $\tau _{\textrm{crit}}=9$ days and vary $\kappa _{\max }$ from 0.6 to 1.4 times its baseline value of $6.4\times 10^{-6}~\mathrm {ml\,hr}^{-1}\textrm{cell}^{-1}$. Stage 2 begins from the operational handoff condition $P(0)=Z_{\textrm{crit}}=5$ and retains the distributed colonized-admission source $\eta \Lambda g_A(\tau )$ with η=0.10. At each immune-strength multiplier, the within-host path and the corresponding transmission and progression kernels are recalculated. The constant transmission rate $\bar{\beta }$ is then selected separately so that the constant and age-dependent profiles have exactly the same $R_0$ within that pair. Therefore, the two curves in panel (a) of Fig. 8 overlap by construction, while $R_0$ varies across the immune-strength scenarios.

Increasing immune strength produces a monotonic but modest reduction in reproductive potential. The paired value of $R_0$ decreases from 0.932383 at 0.6 times baseline $\kappa _{\max }$ to 0.907957 at baseline and 0.893844 at 1.4 times baseline. This corresponds to a 4.133% reduction across the examined range. All values remain below one. Thus, the theoretical importation-free transmission system is subcritical throughout this sensitivity analysis, although the Stage 2 simulations continue to receive colonized admissions through the distributed source.

Panel (b) shows that maximum concurrent total MRSA burden also decreases modestly as immune strength increases. Under age-dependent transmission, the peak decreases from 6.487 patients at 0.6 times baseline to 6.398 patients at baseline and 6.344 patients at 1.4 times baseline. The corresponding matched constant-profile peaks are 6.046, 5.958, and 5.951 patients. At baseline, the age-dependent peak exceeds the matched constant peak by 0.439 patient, or 7.372%. Across the complete immune-strength range, the age-dependent peak decreases by 2.205%, whereas the matched constant peak decreases by 1.586%.

**Fig. 8:**
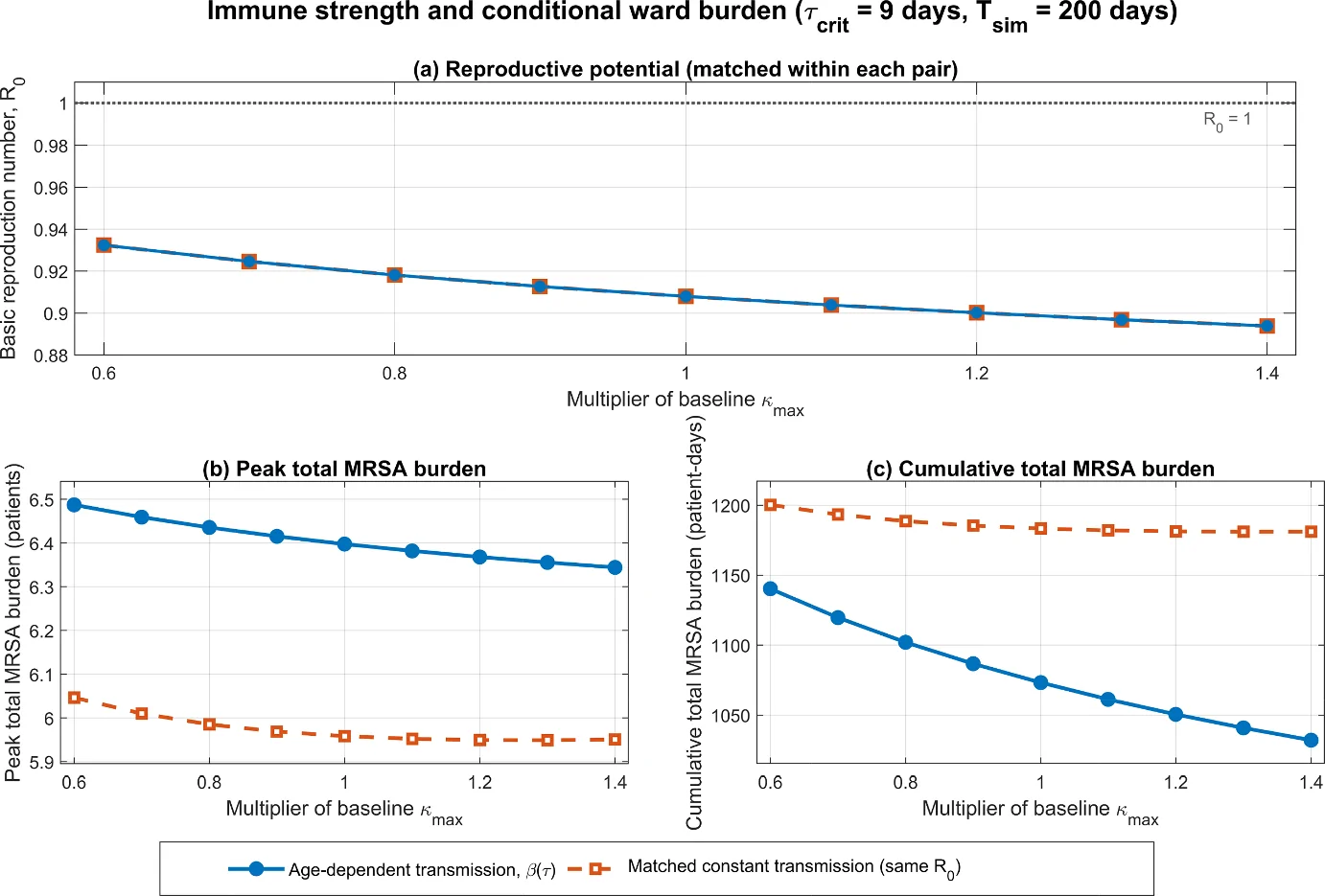
Immune-strength sensitivity under the final distributed-source model. The onset of effective clearance is fixed at $\tau _{\textrm{crit}}=9$ days, while $\kappa _{\max }$ is varied from 0.6 to 1.4 times its baseline value. Stage 2 begins from $P(0)=Z_{\textrm{crit}}=5$, retains the distributed colonized-admission source with η=0.10, and is simulated for $T_{\textrm{sim}}=200$ days. At each multiplier, the constant transmission rate $\bar{\beta }$ is selected separately to give exactly the same $R_0$ as the age-dependent profile. Panel (a) shows the paired reproduction numbers; the two curves overlap because they are matched by construction, and the horizontal dotted line marks $R_0=1$. Panel (b) shows the maximum concurrent total MRSA burden, $\max _{0\le t\le T_{\textrm{sim}}}P(t)$. Panel (c) shows cumulative total MRSA burden, $\int _0^{T_{\textrm{sim}}}P(t)\,dt$, expressed in patient-days, where $P(t)=C_A(t)+C_W(t)+I(t)$. Each plotted point is obtained from a separate paired ward simulation using the representative accepted-median parameters (Color figure online)

Panel (c) shows a clearer difference in cumulative total burden. Under age-dependent transmission, cumulative burden decreases from 1140.3 patient-days at 0.6 times baseline to 1073.2 patient-days at baseline and 1032.0 patient-days at 1.4 times baseline, representing a 9.494% reduction across the examined range. The matched constant-profile values decrease from 1200.3 to 1183.3 and 1181.1 patient-days, representing a 1.593% reduction. At baseline, the age-dependent model produces 110.1 fewer total MRSA patient-days than the matched constant model, a difference of -9.304% relative to the matched constant value. Thus, the age-dependent profile produces a higher short-term peak but a lower burden integrated over the 200-day simulation horizon.

The paired differences are moderate because matching $R_0$ preserves the total survival-weighted transmission potential, while the two models also share the same admission source, patient-flow structure, progression, recovery, decolonization, and mortality processes. Ward turnover and aggregation over $T_{\textrm{sim}}=200$ days further smooth some timing differences. Nevertheless, matching $R_0$ does not match the temporal placement of transmission. For the representative baseline calculation, the age-dependent profile accumulates 50% of $R_0$ by 3.0814 days, compared with 3.8150 days under the matched constant profile. The corresponding 90% times are 6.9833 and 13.0483 days. This earlier concentration is consistent with the higher age-dependent peak and lower long-horizon cumulative burden.

Larger timing-related differences would be expected for transmission functions whose mass is separated more strongly relative to the approximately 5.65-day mean duration of the transmissible states. Examples include a narrow transmission pulse immediately after colonization, a strongly delayed peak near or beyond the typical ICU stay, a long late tail concentrated among the relatively few long-stay patients, or multiple well-separated peaks. Even when such profiles are matched to the same $R_0$, they can produce substantially different generation intervals, patient-level transmission heterogeneity, finite-threshold probabilities, and short-term ward trajectories. The present fitted profile is not this extreme; accordingly, its matched-$R_0$ differences are outcome-dependent and generally moderate rather than uniformly large. These sensitivity calculations use the representative accepted-median parameters and should be interpreted together with the behavioral-set results reported in the preceding subsections.

### Same $R_0$ Does not Imply the Same Operational Outcomes: Matched-$R_0$ Timing-Only Counterfactual

The matched-$R_0$ counterfactual provides a direct test of whether preserving overall reproductive potential is sufficient to preserve the two operational endpoints. The colonization-age-dependent model and its constant-transmission comparator share the same patient-flow structure, admission process, distributed colonized-admission source, progression, recovery, decolonization, and mortality processes. The constant rate $\bar{\beta }$ is selected according to Sect. 2.7 so that the survival-weighted reproduction number is identical to that of the age-dependent profile. Consequently, differences between a matched pair arise from the temporal distribution of transmission opportunity rather than from a difference in $R_0$.

For the representative accepted-median parameter combination, both profiles have $R_0=0.907957$. Nevertheless, their timing and heterogeneity differ. The age-dependent profile accumulates 50% of its reproductive potential by 3.0814 days, whereas the matched constant profile reaches the same cumulative share by 3.8150 days. The corresponding effective dispersion parameters are $k_{\textrm{disp}}^{\textrm{age}}=1.855332$ and $k_{\textrm{disp}}^{\textrm{const}}=1.010124$. Thus, matching the mean transmission potential does not reproduce its temporal placement or its between-individual variability.

The representative Stage 1 finite-threshold comparison is correspondingly close but not identical. At $Z_{\textrm{crit}}=5$, the exact probabilities of reaching the operational handoff threshold before extinction are 0.114659 for the age-dependent profile and 0.117074 for the matched constant profile. The age-dependent-minus-constant difference is -0.002415, or approximately -0.241 percentage points. Moreover, the ordering changes with the selected threshold: the age-dependent probability is higher at $Z_{\textrm{crit}}=2$, 3, and 4, whereas the matched constant probability is slightly higher at the examined thresholds from 5 onward. The complete offspring distribution, rather than $R_0$ or $k_{\textrm{disp}}$ alone, therefore determines the finite-threshold comparison.

The Stage 2 comparison shows a different timing effect. At the baseline values $\tau _{\textrm{crit}}=9$ days and $\kappa _{\max }=6.4\times 10^{-6}~\mathrm {ml\,hr}^{-1}\textrm{cell}^{-1}$, the age-dependent model produces a peak total MRSA burden of 6.398 patients, compared with 5.958 patients under the matched constant profile. This is an age-dependent-minus-constant difference of 0.439 patient, or 7.372%. In contrast, cumulative total burden over $T_{\textrm{sim}}=200$ days is 1073.2 patient-days under age-dependent transmission and 1183.3 patient-days under constant transmission. The corresponding difference is -110.1 patient-days, or -9.304%. Front-loading therefore produces a somewhat higher near-term peak but a lower burden integrated over the full simulation horizon.

**Table 5:** Final representative comparison between the colonization-age-dependent transmission profile and its matched-$R_0$ constant-transmission comparator. The Stage 1 calculations use the importation-free disease-free state.

Outcome	Age-dependent profile	Matched constant profile	Age-dependent minus constant
Basic reproduction number, $R_0$	0.907957	0.907957	0
Time to accumulate 50% of $R_0$, $t_{50}$	3.0814 days	3.8150 days	-0.7336 days
Effective dispersion, $k_{\textrm{disp}}$	1.855332	1.010124	+0.845208
Exact probability of reaching $Z_{\textrm{crit}}=5$	0.114659	0.117074	-0.002415
Peak total MRSA burden	6.398 patients	5.958 patients	+0.439 patient (+7.372%)
Cumulative total MRSA burden	1073.2 patient-days	1183.3 patient-days	-110.1 patient-days (-9.304%)

The Stage 2 burden calculations begin from $P(0)=Z_{\textrm{crit}}=5$, retain the distributed admission source with η=0.10, use $\tau _{\textrm{crit}}=9$ days and baseline $\kappa _{\max }$, and are evaluated over $T_{\textrm{sim}}=200$ days. Percent differences are calculated relative to the matched constant value

Table 5 shows why the importance of timing is outcome-dependent. Matching $R_0$ preserves the expected total number of secondary acquisitions produced at the theoretical disease-free state, but it does not preserve the generation-interval distribution, patient-level transmission heterogeneity, finite-threshold behavior, peak timing, or long-horizon burden. In the fitted profile, the central timing shift is moderate, the representative system is subcritical, and both models experience the same rapid ICU turnover and continuing distributed importation in Stage 2. These shared features limit the magnitude of many paired differences.

Larger differences would require transmission profiles whose reproductive potential is separated more strongly across colonization age relative to patient exit times. Examples include a narrow pulse immediately after colonization, a pronounced delayed peak near or beyond the typical ICU stay, a long late tail concentrated among the relatively few long-stay patients, or multiple well-separated transmission peaks. Such profiles could produce substantially different generation intervals, offspring heterogeneity, finite-threshold probabilities, and transient ward trajectories even when their survival-weighted values of $R_0$ are identical. The fitted profile is not this extreme. The results therefore do not support describing timing as a universally dominant or first-order determinant; rather, timing has an outcome-specific effect whose magnitude and direction depend on the endpoint and operational threshold.

### Synthesis: Implications for the Two Endpoints of the Research Question

For the Stage 1 endpoint, the representative accepted-median transmission profile is subcritical, with $R_0=0.907957$. Ultimate extinction therefore occurs with probability one, and the ultimate non-extinction probability is zero. Nevertheless, a temporary transmission chain can reach the operational handoff threshold before extinction. The exact probability of reaching $Z_{\textrm{crit}}=5$ is 0.114659 under age-dependent transmission and 0.117074 under its matched constant comparator. These are finite-threshold probabilities and must not be interpreted as probabilities of indefinite persistence.

Propagation across all 45, 250 accepted behavioral parameter sets qualifies the representative result. The behavioral median of $R_0$ is 0.917464, with a 95% behavioral range of [0.814593,1.032975]. A total of 92.460% of accepted sets have $R_0<1$, whereas 7.540% have $R_0>1$. At $Z_{\textrm{crit}}=5$, the age-dependent-minus-constant threshold-probability difference has median -0.001060 and behavioral range [-0.007436,0.013802], which crosses zero. Thus, subcriticality is strongly predominant but not universal, and neither the sign nor the magnitude of the finite-threshold timing effect at five is robustly large across accepted fits. By contrast, front-loading is comparatively robust: the age-dependent $t_{50}$ is earlier than the matched constant $t_{50}$ in 98.378% of accepted sets, with a median paired difference of -0.7420 days.

For the Stage 2 endpoint, clearance timing has substantially greater leverage than the examined variation in immune strength. As $\tau _{\textrm{crit}}$ increases from 5 to 15 days, the representative age-dependent value of $R_0$ increases from 0.627881 to 1.078492, peak total MRSA burden increases from 5.629 to 7.298 patients, and cumulative total burden increases from 683.8 to 1398.0 patient-days. In contrast, increasing $\kappa _{\max }$ from 0.6 to 1.4 times baseline while holding $\tau _{\textrm{crit}}=9$ days decreases $R_0$ by only 4.133%, from 0.932383 to 0.893844. Over the same immune-strength range, the age-dependent peak decreases by 2.205%, while cumulative age-dependent burden decreases by 9.494%.

The paired constant-transmission comparisons show that matching $R_0$ does not reproduce every burden summary. At baseline, age-dependent transmission produces a 7.372% higher peak but a 9.304% lower cumulative total burden than the matched constant profile. The model therefore assigns different operational implications to early concentration and prolonged transmission: front-loading can increase near-term ward pressure while a sustained constant rate can produce a larger burden when integrated over a long horizon.

The final fitted regime uses the fixed modeling assumption η=0.10, corresponding to a total colonized-admission rate of $\eta \Lambda =0.30$ patients per day, distributed across pre-admission colonization ages through $g_A(\tau )$. Consequently, Stage 2 burden can persist under continuing external seeding even when the importation-free reproduction number is below one. The practical implication is that the two transmission pathways require complementary controls: admission-focused measures reduce repeated external introductions, while measures that move effective clearance earlier reduce the colonization-age window in which transmission potential is concentrated. Increasing clearance strength without advancing its onset has more limited leverage over reproductive potential in the examined representative regime.

## Conclusion

This study shows that colonization-age timing changes when reproductive potential is accumulated, but its operational consequences are endpoint dependent. For the representative accepted-median parameter combination, the colonization-age-dependent model gives $R_0=0.907957$. It is therefore subcritical at the theoretical importation-free disease-free state, and its ultimate non-extinction probability is zero. Nevertheless, a transmission chain can temporarily reach the operational threshold before becoming extinct. At $Z_{\textrm{crit}}=5$, the exact threshold-reaching probabilities are 0.114659 for the colonization-age-dependent profile and 0.117074 for its matched-$R_0$ constant-transmission comparator. Across the accepted behavioral parameter sets, the median difference, age dependent minus constant, is -0.001060, with a 95% behavioral range of [-0.007436,0.013802]. Because this range crosses zero, the fitted ensemble does not support a large or consistently directed timing effect on the finite-threshold probability at $Z_{\textrm{crit}}=5$.

Propagation across the accepted parameter sets also qualifies the classification based on the representative parameter combination. The behavioral median of $R_0$ is 0.917464, with a 95% behavioral range of [0.814593,1.032975]; 92.460% of accepted sets are below one and 7.540% are above one. Front-loading is more robust than the finite-threshold probability difference: the colonization-age-dependent profile reaches 50% of its reproductive potential earlier than the matched constant profile in 98.378% of accepted sets, with a median difference of -0.741967 days. Thus, the analysis supports robust earlier accumulation of reproductive potential, while uncertainty remains about whether the fitted system is strictly subcritical and about the direction of the small finite-threshold comparison.

Over the ranges examined, the conditional Stage 2 calculations show that clearance timing has greater leverage over ward burden than the examined change in immune strength. When $\tau _{\textrm{crit}}$ is delayed from 5 to 15 days, the representative colonization-age-dependent $R_0$ increases from 0.627881 to 1.078492, the peak concurrent total MRSA burden increases from 5.629 to 7.298 patients, and the 200-day cumulative total MRSA burden increases from 683.8 to 1398.0 patient-days. By comparison, increasing $\kappa _{\max }$ from 0.6 to 1.4 times its baseline value while holding $\tau _{\textrm{crit}}=9$ days reduces $R_0$ from 0.932383 to 0.893844, the peak total burden by 2.205%, and the cumulative total burden by 9.494%; all $R_0$ values in this immune-strength analysis remain below one. These results indicate that shifting effective clearance earlier has greater leverage in this fitted regime than increasing its later maximum intensity over the range examined.

The matched-$R_0$ comparisons further show why the result must be interpreted by endpoint. At the baseline $\tau _{\textrm{crit}}=9$ days, both profiles have $R_0=0.907957$, but 50% of reproductive potential is accumulated by 3.0814 days under the colonization-age-dependent profile and by 3.8150 days under the matched constant profile. The colonization-age-dependent model produces a peak total burden of 6.3975 patients, compared with 5.9583 patients for the constant comparator, a 7.372% increase. In contrast, its 200-day cumulative total burden is 1073.2 patient-days, compared with 1183.3 patient-days for the constant comparator, a 9.304% decrease. Matching $R_0$ therefore preserves total survival-weighted reproductive potential at the disease-free state, but it does not preserve transmission timing, mechanistically induced dispersion, peak concurrent burden, or finite-horizon cumulative burden. The differences are moderate and are not uniformly directed across endpoints.

External seeding must be distinguished from this importation-free interpretation of $R_0$. In the final distributed-source analysis, η=0.10 is a fixed modeling assumption, not an estimate of admission-screening prevalence. With Λ=3 admissions per day, colonized patients enter at a total rate of $\eta \Lambda =0.30$ patients per day, distributed across pre-admission colonization ages by $g_A(\tau )$. Consequently, repeated introductions can maintain ward detections even when the within-ward reproduction number is below one. Because cumulative incidence alone cannot identify the balance between imported colonization and within-ward acquisition, direct admission-screening data and information on acquisition timing would be needed to separate these mechanisms more precisely.

Overall, the two-stage framework separates temporary threshold crossing from indefinite persistence and distinguishes stochastic ignition from conditional ward burden. The findings support evaluating complementary control strategies: reducing colonized admissions lowers external seeding, while interventions intended to shorten the early transmission window include earlier detection, isolation, decolonization, and appropriate therapy. The Stage 2 results describe deterministic conditional trajectories rather than the full distribution of finite-ward stochastic outcomes, and the reported behavioral ranges summarize accepted fits rather than confidence or credible intervals. Future work should combine direct admission-screening data with a capacity-constrained stochastic ward model to quantify these remaining sources of uncertainty.

## Data Availability

The data supporting the findings of this study are available on request from the corresponding author. The data are not publicly available due to privacy or ethical restrictions.

## A Parameter Justification and Derivations (Within-Host and Coupling Components)

This appendix records the rationale and back-of-the-envelope derivations used to set nominal values or plausible ranges for selected parameters in Table 2.

### Intrinsic Bacterial Growth Rate ($r_{\text {bac}}$)

Under favorable conditions, *Staphylococcus aureus* can expand rapidly, with a reported doubling time of approximately $T_d \approx 30$ minutes (0.5 hr) (Pray 2008). For exponential growth, the relationship between doubling time and the continuous-time per-capita growth constant is

$$ r_{\text {bac}}=\frac{\ln (2)}{T_d}. $$

Substituting $T_d=0.5$ hr gives

$$ r_{\text {bac}}=\frac{0.6931}{0.5}\approx 1.386~\text {hr}^{-1}. $$

In the within-host model (5), this represents the maximal per-capita expansion rate in the absence of immune killing and drug-mediated clearance, with growth subsequently tempered by the logistic carrying capacity $K_{\max }$.

### Immune Killing-Rate Transition ($\kappa (\tau )$)

Kinetic data in Ridder et al. (2022) indicate sustained bacterial titers on the order of $10^6$–$10^7$ (reported in CFU per tissue mass) during early infection, followed by a marked clearance phase beginning around approximately 9 days post-infection, coincident with a strong inflammatory and immune shift. To represent a delayed transition from relative immune evasion to active clearance, we model the killing rate as a sigmoidal function of colonization age:

A1 $$\begin{aligned} \kappa (\tau )=\frac{\kappa _{\max }}{1+\exp \{-k_{\kappa }(\tau -\tau _{\textrm{crit}})\}}. \end{aligned}$$

We use $\tau _{\textrm{crit}}=216$ hr (9 days) as the baseline onset time for the clearance transition (Ridder et al. 2022), and explore $k_{\kappa }\in [0.05,0.1]$ $\text {hr}^{-1}$ to represent a comparatively rapid ramp-up once clearance begins. The peak killing magnitude $\kappa _{\max }$ is taken from the same source as a nominal value (Table 2) and is treated as part of the within-host parameterization rather than a uniquely identifiable quantity from ICU-scale data.

### Bacterial Carrying Capacity ($K_{\max }$)

The carrying capacity $K_{\max }$ represents the saturation level of MRSA burden in the local environment of infection and colonization that limits further growth through resource and space constraints. Ridder et al. (2022) report early titers plateauing at approximately $10^6$-$10^7$ in their measurement units. To place the logistic scale on the CFU/ml axis used in (5), we set an order-of-magnitude nominal value

$$ K_{\max }\approx 10^{10}~\text {CFU/ml}, $$

which is consistent with mapping a peak plateau on the order of $10^7$ per tissue mass to a concentration scale and treating the conversion as an approximate normalization. Because $K_{\max }$ primarily sets the saturation level of B(τ), we treat it as a nominal scale parameter and focus inference on downstream coupling parameters (e.g., $B_{50}$ and $\beta _{\max }$) that translate burden into transmission risk.

### Drug-Mediated Bacterial Clearance ($\delta _{\text {drug}}$)

We represent treatment-driven clearance as a first-order decay term $-\delta _{\text {drug}}B(\tau )$ in (5), where $\delta _{\text {drug}}$ is a natural-log removal rate (units $\text {hr}^{-1}$ in the within-host system). We derive a plausible range using time-to-sterilization summaries reported in the BACSARM trial (Pujol et al. 2021). Approximating sterilization as an average $\Delta \log _{10}$ reduction achieved over a mean window *T* (hours), the corresponding natural-log decay constant is

$$ \delta _{\text {drug}} \approx \ln (10)\,\frac{\Delta \log _{10}}{T}. $$

For daptomycin monotherapy, taking $\Delta \log _{10}\approx 3$ over T≈210 hr gives

$$ \delta _{\text {drug}} \approx \ln (10)\,(3/210)\approx 0.033~\text {hr}^{-1}. $$

For daptomycin plus fosfomycin, taking $\Delta \log _{10}\approx 3$ over T≈100 hr gives

$$ \delta _{\text {drug}} \approx \ln (10)\,(3/100)\approx 0.069~\text {hr}^{-1}. $$

When coupling to the ICU-scale model (day units), we convert via $\delta _{\text {drug,day}}=24\,\delta _{\text {drug}}$. These values are used as plausible ranges consistent with improved microbiological outcomes under combination therapy (Pujol et al. 2021).

### Immune-Response Parameters ($\sigma _{\text {imm}},\omega ,\delta _{\text {imm}}$)

To parameterize the immune effector dynamics in (6) without overfitting, we follow the qualitative framework of Pugliese and Gandolfi (2008). We normalize the effector decay rate to $\delta _{\text {imm}}=1~\text {hr}^{-1}$ to fix the within-host time scale and interpret remaining parameters relative to this decay. We set the basal source as a small fraction of the decay-scale replenishment,

$$ \sigma _{\text {imm}} = 0.05\,\delta _{\text {imm}}E_{\text {base}} \quad (\text {cells}\cdot \text {ml}^{-1}\cdot \text {hr}^{-1}), $$

so that, absent bacteria, the system maintains low-level immune surveillance. The stimulation rate ω governs recruitment and activation in proportion to bacterial burden; we use $\omega \in [0.02,\,0.05]$ (Table 2) to allow regimes in which immune amplification can counterbalance bacterial growth, while still permitting prolonged persistence when clearance is delayed.

### Decolonization Rate $r_C(\tau )$ (ICU-Scale, Day Units)

The ward-scale decolonization hazard $r_C(\tau )$ represents the instantaneous clearance rate of colonization at colonization age τ, measured in days to match the ICU model time scale. Following Bootsma et al. (2006) and evidence that intervention effects can wane due to resistance or limited sustained benefit (Huskins et al. 2011), we model

$$ r_C(\tau )=\gamma _{\text {nat}}+\gamma _{\text {int}}e^{-\rho \tau }. $$

We set the natural clearance component to $\gamma _{\text {nat}}=1/370\approx 0.0027~\text {day}^{-1}$ (Bootsma et al. 2006). The intervention component reflects detection and successful treatment: Bootsma et al. report a detection scale of approximately $0.03~\text {day}^{-1}$ and use a decolonization success probability around 0.25, giving

$$ \gamma _{\text {int}}=0.03\times 0.25 = 0.0075~\text {day}^{-1}. $$

The waning parameter ρ is explored in sensitivity analyses (Table 2) to separate robust timing conclusions from uncertainty in sustained intervention effectiveness.
